# The Role of Adiposity in Cardiometabolic Traits: A Mendelian Randomization Analysis

**DOI:** 10.1371/journal.pmed.1001474

**Published:** 2013-06-25

**Authors:** Tove Fall, Sara Hägg, Reedik Mägi, Alexander Ploner, Krista Fischer, Momoko Horikoshi, Antti-Pekka Sarin, Gudmar Thorleifsson, Claes Ladenvall, Mart Kals, Maris Kuningas, Harmen H. M. Draisma, Janina S. Ried, Natalie R. van Zuydam, Ville Huikari, Massimo Mangino, Emily Sonestedt, Beben Benyamin, Christopher P. Nelson, Natalia V. Rivera, Kati Kristiansson, Huei-yi Shen, Aki S. Havulinna, Abbas Dehghan, Louise A. Donnelly, Marika Kaakinen, Marja-Liisa Nuotio, Neil Robertson, Renée F. A. G. de Bruijn, M. Arfan Ikram, Najaf Amin, Anthony J. Balmforth, Peter S. Braund, Alexander S. F. Doney, Angela Döring, Paul Elliott, Tõnu Esko, Oscar H. Franco, Solveig Gretarsdottir, Anna-Liisa Hartikainen, Kauko Heikkilä, Karl-Heinz Herzig, Hilma Holm, Jouke Jan Hottenga, Elina Hyppönen, Thomas Illig, Aaron Isaacs, Bo Isomaa, Lennart C. Karssen, Johannes Kettunen, Wolfgang Koenig, Kari Kuulasmaa, Tiina Laatikainen, Jaana Laitinen, Cecilia Lindgren, Valeriya Lyssenko, Esa Läärä, Nigel W. Rayner, Satu Männistö, Anneli Pouta, Wolfgang Rathmann, Fernando Rivadeneira, Aimo Ruokonen, Markku J. Savolainen, Eric J. G. Sijbrands, Kerrin S. Small, Jan H. Smit, Valgerdur Steinthorsdottir, Ann-Christine Syvänen, Anja Taanila, Martin D. Tobin, Andre G. Uitterlinden, Sara M. Willems, Gonneke Willemsen, Jacqueline Witteman, Markus Perola, Alun Evans, Jean Ferrières, Jarmo Virtamo, Frank Kee, David-Alexandre Tregouet, Dominique Arveiler, Philippe Amouyel, Marco M. Ferrario, Paolo Brambilla, Alistair S. Hall, Andrew C. Heath, Pamela A. F. Madden, Nicholas G. Martin, Grant W. Montgomery, John B. Whitfield, Antti Jula, Paul Knekt, Ben Oostra, Cornelia M. van Duijn, Brenda W. J. H. Penninx, George Davey Smith, Jaakko Kaprio, Nilesh J. Samani, Christian Gieger, Annette Peters, H.-Erich Wichmann, Dorret I. Boomsma, Eco J. C. de Geus, TiinaMaija Tuomi, Chris Power, Christopher J. Hammond, Tim D. Spector, Lars Lind, Marju Orho-Melander, Colin Neil Alexander Palmer, Andrew D. Morris, Leif Groop, Marjo-Riitta Järvelin, Veikko Salomaa, Erkki Vartiainen, Albert Hofman, Samuli Ripatti, Andres Metspalu, Unnur Thorsteinsdottir, Kari Stefansson, Nancy L. Pedersen, Mark I. McCarthy, Erik Ingelsson, Inga Prokopenko, for the European Network for Genetic and Genomic Epidemiology (ENGAGE) consortium

**Affiliations:** 1 Molecular Epidemiology and Science for Life Laboratory, Department of Medical Sciences, Uppsala University, Uppsala, Sweden; 2 Department of Medical Epidemiology and Biostatistics, Karolinska Institutet, Stockholm, Sweden; 3 Wellcome Trust Centre for Human Genetics, University of Oxford, Oxford, United Kingdom; 4 Estonian Genome Center, University of Tartu, Tartu, Estonia; 5 Oxford Centre for Diabetes, Endocrinology and Metabolism, University of Oxford, Oxford, United Kingdom; 6 Institute for Molecular Medicine Finland, University of Helsinki, Helsinki, Finland; 7 deCODE Genetics, Reykjavik, Iceland; 8 Department of Clinical Sciences, Diabetes and Endocrinology, Lund University Diabetes Centre, Lund University and Skåne University Hospital, Malmö, Sweden; 9 Department of Epidemiology, Erasmus Medical Center, Rotterdam, The Netherlands; 10 Department of Biological Psychology, VU University Amsterdam, Amsterdam, The Netherlands; 11 The EMGO Institute for Health and Care Research, Amsterdam, The Netherlands; 12 Institute of Genetic Epidemiology, Helmholtz Zentrum München—German Research Center for Environmental Health, Neuherberg, Germany; 13 Medical Research Institute, Ninewells Hospital and Medical School, University of Dundee, Dundee, United Kingdom; 14 Institute of Health Sciences, University of Oulu, Oulu, Finland; 15 Department of Twin Research and Genetic Epidemiology, King's College London, United Kingdom; 16 Diabetes and Cardiovascular Diseases Genetic Epidemiology Research Unit, Department of Clinical Sciences, Skåne University Hospital, Lund University, Malmö, Sweden; 17 Queensland Institute of Medical Research, Herston, Australia; 18 Queensland Brain Institute, University of Queensland, St Lucia, Australia; 19 Department of Cardiovascular Sciences, University of Leicester, Leicester, United Kingdom; 20 National Institute for Health Research, Leicester Cardiovascular Biomedical Research Unit, Glenfield Hospital, Leicester, United Kingdom; 21 IRCSS Multimedica, Milan, Italy; 22 Institute of Genetics and Biomedical Research, Consiglio Nazionale delle Ricerche, Milan, Italy; 23 Department of Genetic Epidemiology, Erasmus Medical Center, Rotterdam, The Netherlands; 24 Department of Chronic Disease Prevention, National Institute for Health and Welfare, Helsinki, Finland; 25 Public Health Genomics Unit, National Institute for Health and Welfare, Helsinki, Finland; 26 Netherlands Consortium for Healthy Ageing, Netherlands Genomics Initiative, Leiden, The Netherlands; 27 Biocenter Oulu, University of Oulu, Oulu, Finland; 28 Department of Neurology, Erasmus Medical Center, Rotterdam, The Netherlands; 29 Department of Radiology, Erasmus Medical Center, Rotterdam, The Netherlands; 30 Division of Epidemiology, Leeds Institute of Genetics, Health and Therapeutics, School of Medicine, University of Leeds, Leeds, United Kingdom; 31 Institute of Epidemiology I, Helmholtz Zentrum München—German Research Center for Environmental Health, Neuherberg, Germany; 32 Institute of Epidemiology II, Helmholtz Zentrum München—German Research Center for Environmental Health, Neuherberg, Germany; 33 MRC-HPA Centre for Environment and Health, Department of Epidemiology and Biostatistics, School of Public Health, Imperial College London, London, United Kingdom; 34 Department of Obstetrics and Gynaecology, Institute of Clinical Sciences, University of Oulu, Oulu, Finland; 35 Department of Public Health, Hjelt Institute, University of Helsinki, Helsinki, Finland; 36 Institute of Biomedicine, University of Oulu, Oulu, Finland; 37 Department of Psychiatry, Kuopio University Hospital, Kuopio, Finland; 38 Centre for Paediatric Epidemiology and Biostatistics and Medical Research Council Centre for the Epidemiology of Child Health, University College London Institute of Child Health, London, United Kingdom; 39 Hannover Unified Biobank, Hannover Medical School, Hannover, Germany; 40 Research Unit of Molecular Epidemiology, Helmholtz Zentrum München—German Research Center for Environmental Health, Neuherberg, Germany; 41 Department of Social Services and Health Care, Jakobstad, Finland; 42 Folkhälsan Research Centre, Helsinki, Finland; 43 Department of Internal Medicine II–Cardiology, University of Ulm Medical Center, Ulm, Germany; 44 Finnish Institute of Occupational Health, Helsinki, Finland; 45 Steno Diabetes Center, Gentofte, Denmark; 46 Department of Mathematical Sciences, University of Oulu, Oulu, Finland; 47 Wellcome Trust Sanger Institute, Hinxton, United Kingdom; 48 Department of Children, Young People and Families, National Institute for Health and Welfare, Oulu, Finland; 49 Institute of Biometrics and Epidemiology, German Diabetes Center, Düsseldorf University, Düsseldorf, Germany; 50 Department of Internal Medicine, Erasmus Medical Center, Rotterdam, The Netherlands; 51 Institute of Diagnostics, University of Oulu, Oulu, Finland; 52 Department of Internal Medicine, Institute of Clinical Medicine, University of Oulu, Oulu, Finland; 53 Neuroscience Campus Amsterdam, Amsterdam, The Netherlands; 54 Department of Psychiatry, VU University Medical Center, Amsterdam, The Netherlands; 55 Molecular Medicine and Science for Life Laboratory, Department of Medical Sciences, Uppsala University, Uppsala, Sweden; 56 Department of Health Sciences, University of Leicester, Leicester, United Kingdom; 57 Centre for Public Health, Queen's University of Belfast, Belfast, Northern Ireland; 58 Department of Cardiology, Toulouse University School of Medicine, Rangueil Hospital, Toulouse, France; 59 Centre of Excellence for Public Health Northern Ireland, Queen's University of Belfast, Belfast, Northern Ireland; 60 Institute of Cardiometabolism and Nutrition, INSERM UMR S937, Pierre and Marie Curie University, Paris, France; 61 Department of Epidemiology and Public Health, University of Strasbourg, Strasbourg, France; 62 Institut Pasteur de Lille, INSERM U744, Université Lille Nord de France, Lille, France; 63 Epidemiology and Preventive Medicine Research Centre, Department of Clinical and Experimental Medicine, University of Insubria, Varese, Italy; 64 Department of Experimental Medicine, University of Milano-Bicocca, Monza, Italy; 65 Washington University School of Medicine, St Louis, Missouri, United States of America; 66 Population Studies Unit, Department of Chronic Disease Prevention, National Institute for Health and Welfare, Turku, Finland; 67 Department of Health, Functional Capacity and Welfare, National Institute for Health and Welfare, Helsinki, Finland; 68 Department of Clinical Genetics, Erasmus Medical Center, Rotterdam, The Netherlands; 69 Netherlands Genomic Initiative, Leiden, The Netherlands; 70 Centre for Medical Systems Biology, Leiden, The Netherlands; 71 MRC Centre for Integrative Epidemiology Unit, University of Bristol, Bristol, United Kingdom; 72 Department of Mental Health and Substance Abuse Services, National Institute for Health and Welfare, Helsinki, Finland; 73 Munich Heart Alliance, Munich, Germany; 74 Institute of Medical Informatics, Biometry and Epidemiology, Chair of Epidemiology, Ludwig-Maximilians-Universität, Munich, Germany; 75 Klinikum Grosshadern, Munich, Germany; 76 Department of Medicine, Helsinki University Central Hospital, Helsinki, Finland; 77 Department of Medical Sciences, Uppsala University, Uppsala, Sweden; 78 Department of Epidemiology and Biostatistics, Imperial College London, London, United Kingdom; 79 Division of Welfare and Health Promotion, National Institute for Health and Welfare, Helsinki, Finland; 80 Faculty of Medicine, University of Iceland, Reykjavík, Iceland; 81 Oxford National Institute for Health Research Biomedical Research Centre, Churchill Hospital, Oxford, United Kingdom; 82 Department of Genomics of Common Disease, School of Public Health, Imperial College London, London, United Kingdom; Centre for Biomedicine, EURAC, Italy

## Abstract

**Background:**

The association between adiposity and cardiometabolic traits is well known from epidemiological studies. Whilst the causal relationship is clear for some of these traits, for others it is not. We aimed to determine whether adiposity is causally related to various cardiometabolic traits using the Mendelian randomization approach.

**Methods and Findings:**

We used the adiposity-associated variant rs9939609 at the *FTO* locus as an instrumental variable (IV) for body mass index (BMI) in a Mendelian randomization design. Thirty-six population-based studies of individuals of European descent contributed to the analyses.

Age- and sex-adjusted regression models were fitted to test for association between (i) rs9939609 and BMI (*n* = 198,502), (ii) rs9939609 and 24 traits, and (iii) BMI and 24 traits. The causal effect of BMI on the outcome measures was quantified by IV estimators. The estimators were compared to the BMI–trait associations derived from the same individuals. In the IV analysis, we demonstrated novel evidence for a causal relationship between adiposity and incident heart failure (hazard ratio, 1.19 per BMI-unit increase; 95% CI, 1.03–1.39) and replicated earlier reports of a causal association with type 2 diabetes, metabolic syndrome, dyslipidemia, and hypertension (odds ratio for IV estimator, 1.1–1.4; all *p*<0.05). For quantitative traits, our results provide novel evidence for a causal effect of adiposity on the liver enzymes alanine aminotransferase and gamma-glutamyl transferase and confirm previous reports of a causal effect of adiposity on systolic and diastolic blood pressure, fasting insulin, 2-h post-load glucose from the oral glucose tolerance test, C-reactive protein, triglycerides, and high-density lipoprotein cholesterol levels (all *p*<0.05). The estimated causal effects were in agreement with traditional observational measures in all instances except for type 2 diabetes, where the causal estimate was larger than the observational estimate (*p* = 0.001).

**Conclusions:**

We provide novel evidence for a causal relationship between adiposity and heart failure as well as between adiposity and increased liver enzymes.

*Please see later in the article for the Editors' Summary*

## Abstract 2

In this study, Prokopenko and colleagues provide novel evidence for causal relationship between adiposity and heart failure and increased liver enzymes using a Mendelian randomization study design.

*Please see later in the article for the Editors' Summary*

## Editors' Summary

Cardiovascular disease (CVD)—disease that affects the heart and/or the blood vessels—is a major cause of illness and death worldwide. In the US, for example, coronary heart disease—a CVD in which narrowing of the heart's blood vessels by fatty deposits slows the blood supply to the heart and may eventually cause a heart attack—is the leading cause of death, and stroke—a CVD in which the brain's blood supply is interrupted—is the fourth leading cause of death. Globally, both the incidence of CVD (the number of new cases in a population every year) and its prevalence (the proportion of the population with CVD) are increasing, particularly in low- and middle-income countries. This increasing burden of CVD is occurring in parallel with a global increase in the incidence and prevalence of obesity—having an unhealthy amount of body fat (adiposity)—and of metabolic diseases—conditions such as diabetes in which metabolism (the processes that the body uses to make energy from food) is disrupted, with resulting high blood sugar and damage to the blood vessels.

**Why Was This Study Done?:**

Epidemiological studies—investigations that record the patterns and causes of disease in populations—have reported an association between adiposity (indicated by an increased body mass index [BMI], which is calculated by dividing body weight in kilograms by height in meters squared) and cardiometabolic traits such as coronary heart disease, stroke, heart failure (a condition in which the heart is incapable of pumping sufficient amounts of blood around the body), diabetes, high blood pressure (hypertension), and high blood cholesterol (dyslipidemia). However, observational studies cannot prove that adiposity causes any particular cardiometabolic trait because overweight individuals may share other characteristics (confounding factors) that are the real causes of both obesity and the cardiometabolic disease. Moreover, it is possible that having CVD or a metabolic disease causes obesity (reverse causation). For example, individuals with heart failure cannot do much exercise, so heart failure may cause obesity rather than vice versa. Here, the researchers use “Mendelian randomization” to examine whether adiposity is causally related to various cardiometabolic traits. Because gene variants are inherited randomly, they are not prone to confounding and are free from reverse causation. It is known that a genetic variant (rs9939609) within the genome region that encodes the fat-mass- and obesity-associated gene (*FTO*) is associated with increased BMI. Thus, an investigation of the associations between rs9939609 and cardiometabolic traits can indicate whether obesity is causally related to these traits.

**What Did the Researchers Do and Find?:**

The researchers analyzed the association between rs9939609 (the “instrumental variable,” or IV) and BMI, between rs9939609 and 24 cardiometabolic traits, and between BMI and the same traits using genetic and health data collected in 36 population-based studies of nearly 200,000 individuals of European descent. They then quantified the strength of the causal association between BMI and the cardiometabolic traits by calculating “IV estimators.” Higher BMI showed a causal relationship with heart failure, metabolic syndrome (a combination of medical disorders that increases the risk of developing CVD), type 2 diabetes, dyslipidemia, hypertension, increased blood levels of liver enzymes (an indicator of liver damage; some metabolic disorders involve liver damage), and several other cardiometabolic traits. All the IV estimators were similar to the BMI–cardiovascular trait associations (observational estimates) derived from the same individuals, with the exception of diabetes, where the causal estimate was higher than the observational estimate, probably because the observational estimate is based on a single BMI measurement, whereas the causal estimate considers lifetime changes in BMI.

**What Do These Findings Mean?:**

Like all Mendelian randomization studies, the reliability of the causal associations reported here depends on several assumptions made by the researchers. Nevertheless, these findings provide support for many previously suspected and biologically plausible causal relationships, such as that between adiposity and hypertension. They also provide new insights into the causal effect of obesity on liver enzyme levels and on heart failure. In the latter case, these findings suggest that a one-unit increase in BMI might increase the incidence of heart failure by 17%. In the US, this corresponds to 113,000 additional cases of heart failure for every unit increase in BMI at the population level. Although additional studies are needed to confirm and extend these findings, these results suggest that global efforts to reduce the burden of obesity will likely also reduce the occurrence of CVD and metabolic disorders.

**Additional Information:**

Please access these websites via the online version of this summary at http://dx.doi.org/10.1371/journal.pmed.1001474.

- The American Heart Association provides information on all aspects of cardiovascular disease and  tips on keeping the heart healthy, including weight management (in several languages); its website includes personal stories about stroke and heart attacks
- The US Centers for Disease Control and Prevention has information on heart disease, stroke, and all aspects of overweight and obesity (in English and Spanish)
- The UK National Health Service Choices website provides information about cardiovascular disease and obesity, including a personal story about losing weight
- The World Health Organization provides information on obesity (in several languages)
- The International Obesity Taskforce provides information about the global obesity epidemic
- Wikipedia has a page on Mendelian randomization (note: Wikipedia is a free online encyclopedia that anyone can edit; available in several languages)
- MedlinePlus provides links to other sources of information on heart disease, on vascular disease, on obesity, and on metabolic disorders (in English and Spanish)
- The International Association for the Study of Obesity provides maps and information about obesity worldwide
- The International Diabetes Federation has a web page that describes types, complications, and risk factors of diabetes

## Introduction

The incidence and prevalence of cardiovascular disease (CVD) are continuously increasing in parallel with the increase in obesity and metabolic diseases, especially in low- and middle-income countries [1]. An association between increased body mass index (BMI) and cardiometabolic diseases has been demonstrated by many well-designed epidemiological studies, and has previously been shown to be close to log-linear, at least for BMI>25 kg/m^2^ [2]. However, confounding, reverse causation, and other issues with conventional observational studies can seriously impair the possibility of making causal inference, and lead to imprecision in estimation of both the direction and magnitude of the effects, as has been shown for the associations between BMI and mortality from respiratory disease and lung cancer [3]. Several randomized clinical trials have found that lifestyle interventions aiming at weight loss decrease the risk of type 2 diabetes (T2D) and metabolic syndrome [4]–[6], whereas the follow-ups of these studies for CVD outcomes have been underpowered [7],[8]. The causal relationships of long-term obesity to disease are difficult to assess within conventional randomized clinical trials, necessitating other study designs.

**Figure 1:**
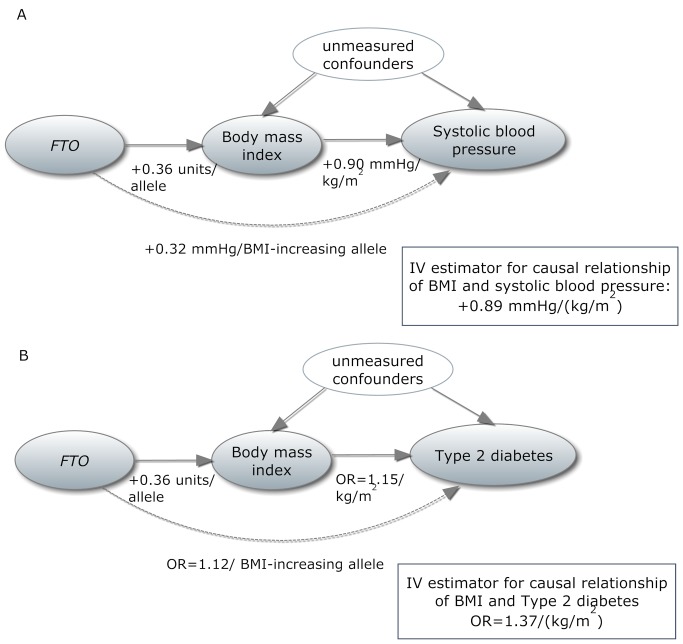
In a Mendelian randomization framework, genotype–phenotype association is assumed to be independent of confounding factors. (A) In an example from our study, the IV estimator is calculated as the beta coefficient from the association of *FTO* with systolic blood pressure divided by the beta coefficient from the association of *FTO* with BMI (IV estimator = 0.32/0.36 = 0.89 mm Hg/BMI unit). The IV estimator is equivalent to what is seen when systolic blood pressure is regressed on BMI. These results are supportive of a causal, non-confounded relationship. For binary traits, the calculation of the IV estimator is done on the log-odds scale. (B) The relationship of BMI with T2D, where the IV estimator is ln(OR_IV_) = ln(1.12)/0.36, which equals a causal OR of BMI for T2D of 1.37. This is larger than what is seen in the standard age- and sex-adjusted logistic regression of T2D on BMI (*p* = 0.001), indicating that confounding or reverse causation may be present or that BMI measured once in adulthood does not fully reflect the effect of lifetime adiposity.

In the past decade, instrumental variable (IV) analysis has become widely used for assessing causality using genetic variants under the name of “Mendelian randomization” (MR) [9]. MR represents one of the methods to infer causal relationships between epidemiologically relevant phenotypes. In MR study designs, a genetic variant associated with an intermediate phenotype (in the present report, BMI) is used as an IV to evaluate the causal relationship of the intermediate phenotype with the outcome of interest (Figure 1). Since genetic variants are assumed to be randomly distributed within a population, the IV is regarded as independent of confounders affecting the intermediate phenotype (BMI)–outcome relationship [10]. In the presence of confounding and reverse causation, the IV approach is an alternative for statistical estimation of causal relationships, especially within large-scale studies, where classical epidemiological modeling—fully adjusted for a wide range of covariates and across numerous outcomes—would be difficult. While acknowledging the issue of observed and unobserved confounding, we consider MR as a pragmatic tool for elucidating the epidemiological data through utilization of the findings from genetic association studies on intermediate phenotypes. The strength of the causal interpretation depends crucially on the validity of assumptions and caveats within MR experiments, some of which are difficult to evaluate [11]. If the basic assumptions are violated, invalid conclusions would be drawn from the experiments. In the past five years, large-scale collaborative efforts have successfully identified more than 30 loci associated with BMI and obesity [12]. The single nucleotide polymorphism (SNP) rs9939609, within the fat-mass- and obesity-associated gene (*FTO*) locus, was the first associated with BMI by genome-wide association studies, and the association has been extensively replicated in individuals of European descent and in other ethnic groups [12]. *FTO* locus variants alone have been reported to explain 0.34% of the phenotypic variability in BMI [13], and the rs9939609 variant is considered a good instrument in MR studies because of its specificity (lack of known pleiotropy) and decent effect size [14],[15].

Several MR studies using *FTO* variants have supported the hypothesis of a causal relationship between adiposity and cardiometabolic phenotypes, such as ischemic heart disease, C-reactive protein (CRP), systolic and diastolic blood pressure, fasting insulin, triglycerides, metabolic syndrome, and decreased concentrations of high-density lipoprotein cholesterol (HDL-C) [14]–[19]. However, the causal relationship between obesity and increased risk of other CVD and metabolic phenotypes, such as heart failure, stroke, and non-alcoholic fatty liver disease, is not yet established using these methods, probably because of power issues, as large sample sizes are needed for MR studies [15]. Table 1 shows an overview of previous MR studies of adiposity and cardiometabolic phenotypes, with reported sample sizes and instruments used.

**Table 1:** Comparison of our study with previous Mendelian randomization studies of adiposity on cardiometabolic phenotypes.

Phenotype	Present Study Using *FTO* as Instrument			Previous Studies				
	*N* Total	*N* Cases	Evidence for Causality?	*N* Total	*N* Cases	Evidence for Causality?	Reference	Instrument Other than *FTO* Only
CHD	119,630	10,372	−	75,627	11,056	+	[16]	*FTO, MC4R, TMEM18*
Heart failure	75,770	6,068	+				N.A.	
Hemorrhagic stroke	77,020	588	−				N.A.	
Ischemic stroke	106,402	4,233	−				N.A.	
Stroke	85,175	4,003	−				N.A.	
T2D	160,347	20,804	+				—a	
Dyslipidemia	96,380	33,414	+				N.A.	
Hypertension	155,191	56,721	+	37,027	24,813	+	[18]	*FTO, MC4R*
Metabolic syndrome	49,592	11,608	+	12,555	N.A.	+	[15]	
Mortality	68,762	8,640	−				N.A.	
2-h post-OGTT glucose	21,257		+				N.A.	
Fasting glucose	84,910		−	13,632		+	[15]	
				2,230		+	[17]	
HbA1c	35,471		−	8,876		−	[15]	
Fasting insulin	48,018		+	12,095		+	[15]	
				2,229		−	[17]	
Diastolic blood pressure	130,380		+	15,619		−	[15]	
				37,010		+	[18]	*FTO, MC4R*
Systolic blood pressure	147,644		+	15,624		−	[15]	
				37,011		+	[18]	*FTO, MC4R*
				2,204		+	[17]	
HDL-C	132,782		+	13,659		+	[15]	
				2,224		−	[17]	
LDL-C	123,026		−	13,476		−	[15]	
				2,224		−	[17]	
ALT	46,754		+	6,171		−	[15]	
CRP	91,337		+	21,836		+	[18]	
				2,133		−	[17]	
				5,804		+	[19]	*FTO, MC4R*
GGT	71,118		+	6,596		−	[15]	
IL-6	11,225		−				N.A.	
Triglycerides	139,241		+	13,651		+	[15]	
				2,228		−	[17]	
Total cholesterol	147,619		−	2,226		−	[17]	

a No formal MR study, although the association of *FTO* and T2D is well known.

N.A, not applicable.

In the present investigation, which is the largest MR study to date, we aimed to evaluate the evidence for a causal relationship between adiposity, assessed as elevated BMI, and a wide range of cardiometabolic phenotypes including coronary heart disease, stroke, T2D, and heart failure, as well as a number of intermediate phenotypes related to future disease end points.

## Methods

The study was conducted within the European Network for Genetic and Genomic Epidemiology (ENGAGE) consortium, represented here by 36 cross-sectional and longitudinal cohort studies and up to 198,502 individuals of European descent (Table S1).

### Genotypes

Of the many highly correlated variants within the *FTO* locus, we chose the widely confirmed and extensively studied variant rs9939609 as the index SNP and IV for this study. Whenever possible, we used direct genotype information for rs9939609 from participating cohorts (*n* = 21) that had *FTO* variant genotypes available (Table S2). Eleven out of 36 studies performed de novo genotyping of rs9939609 for the present study, and ten studies used direct genotype information on rs9939609 from previously genotyped array data. Whenever rs9939609 was not genotyped directly, we used either (i) the HapMap II CEU (European) reference panel–imputed genetic information from genome-wide association studies (http://hapmap.ncbi.nlm.nih.gov/downloads/genotypes/2008-10_phaseII/) for rs9939609 (*n* = 5) or (ii) genotype information from a predefined list of proxies that are in high linkage disequilibrium (LD) with rs9939609 (*n* = 10, *r* ^2^>0.9; Table S3). For the remaining studies, we used the directly genotyped proxies rs11075989 (*n* = 5, *r* ^2^ = 1.0), rs3751812 (*n* = 4, *r* ^2^ = 1.0), and rs1421085 (*n* = 1, *r* ^2^ = 0.93). We estimated effects of the BMI-increasing A allele of rs9939609, or for the corresponding alleles from proxies (using HapMap II CEU LD data), on phenotypes. We excluded individuals from analysis when the overall array sample call rate was <95%. All studies reported SNPs with Hardy-Weinberg equilibrium exact *p*>0.0001, an information content >0.99 for imputed SNPs, and a call rate>0.95 for genotyped SNPs.

### Outcomes

We studied nine dichotomous cardiometabolic outcomes in up to 160,347 individuals and 14 quantitative cardiometabolic traits in up to 147,644 individuals. Only individuals with both BMI and *FTO* genotype information available were included in the study.

The CVD dichotomous outcomes of interest were coronary heart disease (CHD), heart failure, hemorrhagic stroke, ischemic stroke, all-cause stroke, and hypertension diagnosed at any time point (ever) during the life course (Table 2). The metabolic dichotomous outcomes included dyslipidemia, metabolic syndrome, and T2D diagnosed at any time point (ever) during the life course. The diagnoses of CHD, heart failure, hemorrhagic stroke, ischemic stroke, all-cause stroke, and all-cause mortality were based on health registries and/or validated medical records (Table S4). Hypertension, dyslipidemia, and T2D diagnoses could be self-reported or based on biochemical measurement within the study, in addition to health registries and validated medical records (Table S4). The diagnosis of metabolic syndrome was based on a modified National Cholesterol Education Program Adult Treatment Panel III definition [20]. We analyzed a subset of individuals with prospectively collected events available for incident cases of all binary outcomes and for all-cause mortality as outcome.

**Table 2:** Meta-analysis results of Mendelian randomization analyses on effect of *FTO*-derived adiposity on cardiovascular and metabolic disease: dichotomous outcomes.

Dichotomous Outcomes	Number of Studies	Number of Cases	Number of Controls	BMI–Trait^a^		*FTO*–Trait^b^		IV Estimator^a^		Difference IV/BMI–Trait *p*-Value
				OR/HR (95% CI)	*p*-Value	OR/HR (95% CI)	*p*-Value	OR/HR (95% CI)	*p*-Value	
Ever CHD	19	10,372	109,258	1.030 (1.012, 1.048)	0.001	0.998 (0.955, 1.043)	0.94	0.995 (0.879, 1.126)	0.94	0.59
Incident CHD	11	3,482	47,165	1.046 (1.031, 1.061)	8.3×10^−10^	0.995 (0.948, 1.044)	0.83	0.986 (0.861, 1.129)	0.83	0.39
Ever heart failure	13	6,068	69,702	1.085 (1.060, 1.111)	1.1×10^−11^	1.058 (1.016, 1.102)	0.006	1.173 (1.044, 1.318)	0.007	0.20
Incident heart failure	9	2,863	44,400	1.097 (1.080, 1.115)	3.5×10^−29^	1.064 (1.009, 1.122)	0.02	1.191 (1.025, 1.385)	0.023	0.29
Ever hemorrhagic stroke	8	588	76,432	0.987 (0.959, 1.016)	0.37	0.985 (0.861, 1.126)	0.82	0.957 (0.657, 1.396)	0.82	0.87
Incident hemorrhagic stroke	6	280	19,721	0.988 (0.939, 1.041)	0.66	0.865 (0.693, 1.080)	0.20	0.666 (0.356, 1.245)	0.20	0.21
Ever ischemic stroke	13	4,233	102,169	1.024 (1.004, 1.044)	0.017	0.992 (0.944, 1.042)	0.75	0.978 (0.851, 1.124)	0.75	0.52
Incident ischemic stroke	11	1,617	47,085	1.034 (1.013, 1.056)	0.001	1.033 (0.955, 1.117)	0.42	1.095 (0.877, 1.367)	0.42	0.61
Ever stroke	18	4,003	81,172	1.012 (0.994, 1.030)	0.20	0.997 (0.950, 1.046)	0.90	0.992 (0.866, 1.136)	0.90	0.78
Incident stroke	11	2,473	46,140	1.024 (1.008, 1.040)	0.003	1.016 (0.951, 1.085)	0.64	1.045 (0.868, 1.258)	0.64	0.83
Ever T2D	28	20,804	139,543	1.151 (1.135, 1.168)	5.6×10^−85^	1.117 (1.081, 1.155)	6.7×10^−11^	1.366 (1.234, 1.513)	2.0×10^−9^	0.001
Incident T2D	6	1,991	29,264	1.160 (1.142, 1.178)	1.7×10^−75^	1.112 (1.044, 1.184)	0.001	1.347 (1.123, 1.616)	0.001	0.19
Ever dyslipidemia	24	33,414	62,966	1.150 (1.128, 1.172)	1.3×10^−45^	1.047 (1.026, 1.068)	1.14×10^−5^	1.138 (1.072, 1.209)	2.6×10^−5^	0.76
Incident dyslipidemia	1^c^	237	360	1.059 (1.013, 1.107)	0.01	1.036 (0.858, 1.250)	0.72	1.104 (0.648, 1.884)	0.72	0.88
Ever hypertension	27	56,721	98,470	1.126 (1.114, 1.139)	2.5×10^−100^	1.044 (1.025, 1.063)	2.6×10^−6^	1.128 (1.070, 1.189)	7.0×10^−6^	0.95
Incident hypertension	1^c^	600	137	1.042 (1.012, 1.072)	5.5×10^−3^	1.032 (0.917, 1.161)	0.60	1.093 (0.783, 1.527)	0.60	0.78
Ever metabolic syndrome	16	11,608	37,984	1.321 (1.282, 1.361)	1.1×10^−73^	1.099 (1.063, 1.137)	3.96×10^−8^	1.309 (1.182, 1.450)	2.6×10^−7^	0.87
Incident metabolic syndrome	1^c^	458	641	1.209 (1.173, 1.245)	4.2×10^−36^	1.134 (0.995, 1.292)	0.06	1.428 (0.982, 2.076)	0.06	0.38
Incident mortality	13	8,640	60,122	1.015 (1.001, 1.030)	0.04	0.994 (0.964, 1.025)	0.69	0.983 (0.902, 1.071)	0.69	0.47

a OR/HR corresponds to one-unit increase in BMI (kg/m^2^).

b OR/HR corresponds to per-allele change.

c Only one study; meta-analysis not performed.

HR, hazard ratio.

We studied the following quantitative phenotypes (Table 3): (i) measurements of glucose homeostasis in individuals without diabetes: fasting glucose, 2-h post-load glucose from the oral glucose tolerance test (OGTT), hemoglobin A1c (HbA1c), and fasting insulin; (ii) diastolic and systolic blood pressure, with adjustment for blood pressure medication; (iii) lipid metabolism (in individuals without lipid-lowering medication): HDL-C, low-density lipoprotein cholesterol (LDL-C), total cholesterol, and triglycerides; (iv) liver enzyme activity and leakage: alanine aminotransferase (ALT) and gamma-glutamyl transferase (GGT); and (v) inflammation markers: CRP and interleukin-6 (IL-6). Prior to analysis the following variables were transformed to the natural logarithmic scale: fasting insulin, ALT, GGT, CRP, IL-6, and triglycerides (assay specifications are reported in Table S5).

**Table 3:** Meta-analysis results of Mendelian randomization analyses on effect of *FTO*-derived adiposity on cardiovascular and metabolic disease: quantitative phenotypes.

Quantitative Phenotypes	Units	Number of Studies	*N*	BMI–Trait^a^		FTO–Trait^b^		IV Estimator^a^		Difference IV/BMI–Trait *p*-Value
				β (95% CI)	*p*-Value	β (95% CI)	*p*-Value	β (95% CI)	*p*-Value	
2-h post-OGTT glucose	mmol/l	8	21,257	0.062 (0.037, 0.087)	1.1×10^−6^	0.031 (0.005, 0.057)	0.02	0.088 (0.013, 0.163)	0.02	0.51
Fasting glucose	mmol/l	22	84,910	0.028 (0.024, 0.033)	4.0×10^−34^	0.006 (−0.002, 0.014)	0.12	0.018 (−0.005, 0.040)	0.12	0.36
HbA1c	%	12	35,471	0.022 (0.014, 0.029)	6.3×10^−9^	0.002 (−0.005, 0.010)	0.49	0.007 (−0.013, 0.027)	0.49	0.19
Fasting insulin^c^	pmol/l	17	48,018	0.060 (0.055, 0.065)	1.3×10^−135^	0.020 (0.013, 0.027)	5.54×10^−9^	0.056 (0.036, 0.077)	5.7×10^−8^	0.74
Diastolic blood pressure	mm Hg	29	130,380	0.619 (0.554, 0.685)	3.0×10^−76^	0.174 (0.069, 0.280)	0.001	0.490 (0.187, 0.793)	0.002	0.41
Systolic blood pressure	mm Hg	30	147,644	0.903 (0.807, 0.999)	6.7×10^−76^	0.317 (0.175, 0.460)	1.3×10^−5^	0.892 (0.475, 1.309)	2.8×10^−5^	0.97
HDL-C	mmol/l	34	132,782	−0.022 (−0.024, −0.021)	4.6×10^−116^	−0.006 (−0.009, −0.003)	1.4×10^−5^	−0.018 (−0.026, −0.009)	3.9×10^−5^	0.28
LDL-C	mmol/l	33	123,026	0.018 (0.013, 0.023)	7.9×10^−14^	0.004 (−0.004, 0.012)	0.35	0.011 (−0.012, 0.035)	0.35	0.59
ALT^c^	U/l	11	46,754	0.027 (0.020, 0.033)	3.1×10^−15^	0.012 (0.006, 0.018)	1.21×10^−4^	0.034 (0.016, 0.052)	2.0×10^−4^	0.43
CRP^c^	mg/l	20	91,337	0.081 (0.061, 0.101)	6.8×10^−16^	0.024 (0.013, 0.035)	4.37×10^−5^	0.068 (0.034, 0.102)	8.1×10^−5^	0.52
GGT^c^	U/l	15	71,118	0.032 (0.028, 0.036)	2.2×10^−51^	0.013 (0.007, 0.019)	3.42×10^−5^	0.037 (0.019, 0.055)	6.6×10^−5^	0.60
IL-6^c^	pg/ml	7	11,225	0.034 (0.027, 0.041)	3.9×10^−21^	0.002 (−0.018, 0.022)	0.87	0.005 (−0.052, 0.062)	0.87	0.32
Triglycerides^c^	mmol/l	34	139,241	0.034 (0.032, 0.036)	4.0×10^−201^	0.010 (0.006, 0.014)	1.44×10^−6^	0.029 (0.016, 0.041)	4.6×10^−6^	0.43
Total cholesterol	mmol/l	34	147,619	0.016 (0.011, 0.021)	2.5×10^−11^	0.002 (−0.006, 0.011)	0.62	0.006 (−0.018, 0.030)	0.63	0.41

a Beta coefficient corresponds to one-unit increase in BMI (kg/m^2^).

b Beta coefficient corresponds to per-allele change.

c Values were transformed to natural logarithm scale prior to analysis.

### Statistical Analyses

Association analyses. We assessed associations between the dichotomous outcomes and (i) *FTO* and (ii) BMI in each cohort using sex- and age-adjusted logistic regression models. We used Cox proportional hazards models to assess *FTO* and BMI associations with prospectively collected events [21]. The time origin in the present analysis was set to the date of first BMI measurement available. We assumed log-additive genetic effects on binary traits. We evaluated the associations of (i) *FTO* and (ii) BMI with the quantitative traits, as well as the association between *FTO* and BMI, using sex- and age-adjusted linear regression in each cohort, assuming an additive effect of the number of A alleles. The models are described in detail in Text S1. The software used for statistical analysis within each cohort is listed in Table S1.

Meta-analyses. As initial attempts at fixed-effects inverse-variance-weighted meta-analysis indicated considerable between-cohort heterogeneity, we performed random-effects meta-analyses, leading to essentially unchanged effect estimates, but somewhat more conservative confidence intervals (Figure S1). Hence, all results presented are from random-effects meta-analysis. Analyses were run at two centers in parallel using different software packages (GWAMA and R) [22],[23] and yielding identical results.

Instrumental variable analyses. We used the IV estimators to quantify the strength of the causal association between BMI and cardiometabolic traits. The estimate was found as a ratio between the two regression coefficients determined from association meta-analyses (Equation 1): estimated *FTO* effect on the given trait and estimated *FTO* effect on BMI in the full study sample (*n* = 198,502). For binary traits, the formula is identical to the Wald estimator [24].
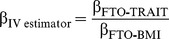
(1)


For quantitative and binary outcomes with only one SNP as instrument, the IV estimator derived by Equation 1 is identical to that derived by the widely used two-stage least squares method [25]. The standard errors for the IV estimators were estimated using the delta method (Equation 2), ignoring correlation, based on a comprehensive sensitivity analysis; see Text S1, Figure S2, and Tables S6 and Table S7 for further details.
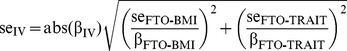
(2)


For each trait, we tested the null hypothesis of no difference between the respective IV estimator and the conventional regression-based estimator of the effect of BMI on trait via a classical *z*-test.

We did not apply correction for multiple testing as the associations between BMI and multiple cardiometabolic traits are widely reported [2],[5].

## Results

### Association between *FTO* Variant and BMI

**Figure 2:**
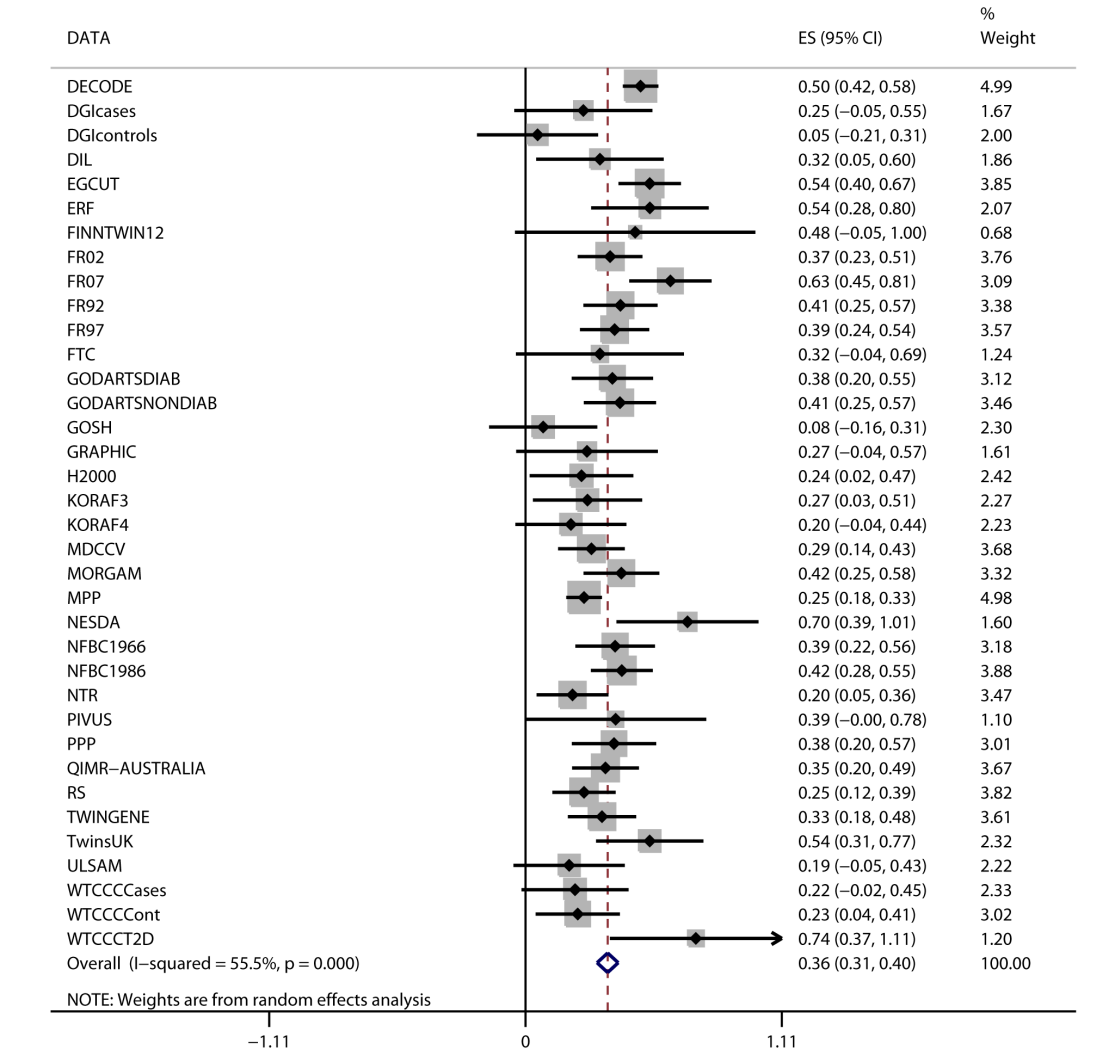
Association between *FTO* variant rs9939609 and BMI in 198,502 individuals. The assigned weight for each study in the meta-analysis is shown in percent (% Weight). ES, estimate. For cohort abbreviations and references, see Table S1.

Random-effects meta-analysis of the association between *FTO* variant and BMI in the 36 studies (*n* = 198,502) showed a positive effect of the A allele on BMI (β = 0.36 per additional A allele; 95% CI, 0.31–0.40; *p* = 4.3×10^−52^), with an effect size in line with that of previous studies [13]. The effect estimates ranged between 0.05 and 0.74 BMI units per copy of A allele, yielding an *I* ^2^ for heterogeneity between studies of 55% (*p* = 3.6×10^−5^; Figure 2; Text S1). We assessed potential causes of this heterogeneity in a meta-regression of the study-specific beta coefficient estimates of effect sizes for the association between *FTO* and BMI—including study-specific mean age and mean BMI as covariates—and whether the study was exclusively of a diabetes case group or not. Effect size estimates decreased non-significantly with increasing cohort age in cohorts with mean age>40 y (*n* = 31, *p* = 0.07).

### Associations between BMI and Cardiometabolic Traits

**Figure 3:**
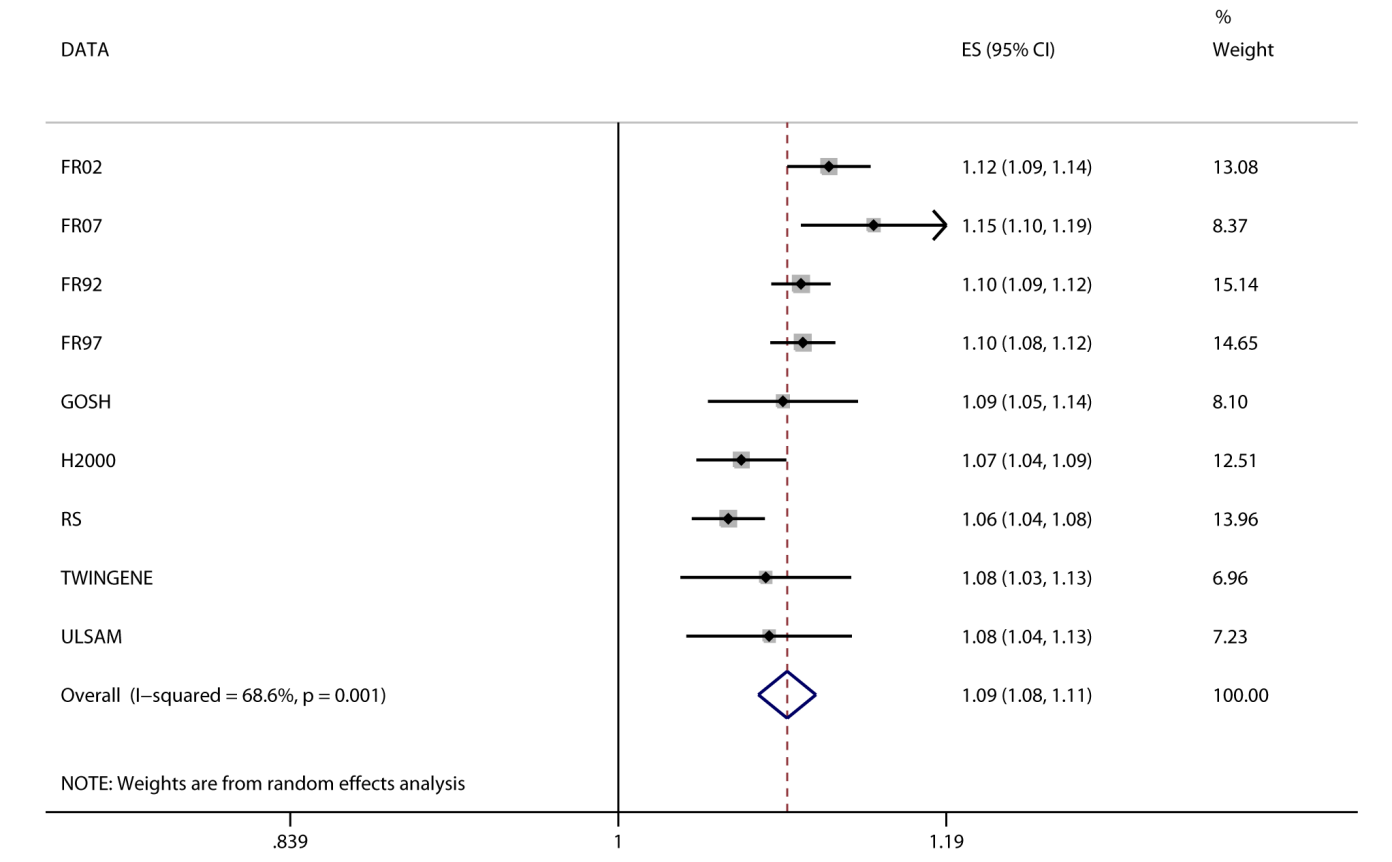
Association between BMI and incident heart failure in 2,863 cases and 44,400 controls. Estimates (ES) are shown on a hazard ratio scale for a one-unit increase in BMI. The assigned weight for each study in the meta-analysis is shown in percent (% Weight). For cohort abbreviations and references, see Table S1.

We observed positive associations (all *p*<0.05) between BMI and ever and incident heart failure (Figure 3), ever and incident CHD, ever all-cause stroke, ischemic stroke, hypertension, dyslipidemia, metabolic syndrome, T2D, and mortality (Table 2). We did not observe an association between BMI and ever or incident hemorrhagic stroke, or incident all-cause stroke. BMI was associated (all *p*<10^−6^) with all quantitative phenotypes: (i) fasting glucose, fasting insulin, 2-h post-OGTT glucose, and HbA1c; (ii) diastolic and systolic blood pressure; (iii) HDL-C, LDL-C, total cholesterol, and triglycerides; (iv) ALT and GGT; and (v) CRP and IL-6 (Table 3).

### Associations between *FTO* Variant and Cardiometabolic Traits

**Figure 4:**
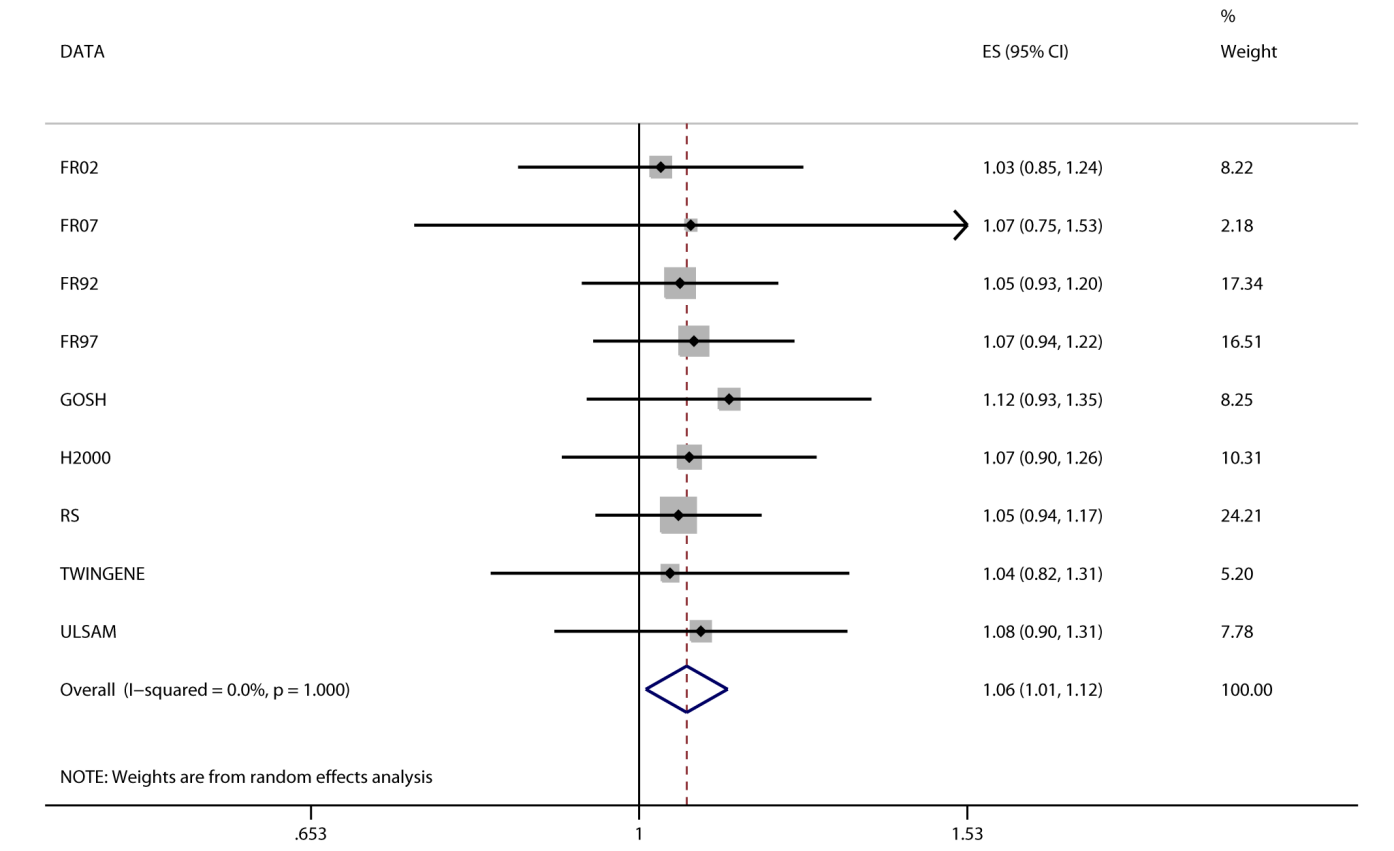
Association between *FTO* and incident heart failure in 2,863 cases and 44,400 controls. Estimates (ES) are shown on a hazard ratio scale per number of effect alleles. The assigned weight for each study in the meta-analysis is shown in percent (% Weight). For cohort abbreviations and references, see Table S1.

We detected a novel association between the BMI-increasing allele of the *FTO* variant and increased odds/hazard ratios of ever and incident heart failure (Figure 4;Table 2). Associations (all *p*<0.001) were observed between the *FTO* variant and increased odds/hazard ratios of ever or incident T2D, ever dyslipidemia, ever metabolic syndrome, and ever hypertension. The *FTO* variant was associated (all *p*<0.05) with increased levels of 2-h post-OGTT glucose, fasting insulin, diastolic blood pressure, systolic blood pressure, triglycerides, ALT, GGT, CRP, and decreased HDL-C.

### Instrumental Variable Analysis

We identified at least nominally significant (*p*<0.05) causal estimates for the effect of BMI (IV estimators) on ever and incident heart failure, ever hypertension, ever and incident T2D, ever dyslipidemia, and ever metabolic syndrome (Table 2). For other dichotomous outcomes, we were not able to confirm the presence of a causal effect of BMI using the IV approach. The estimates derived from IV analysis based on either logistic regression modeling or Cox proportional hazards models were similar for our significant findings.

The IV estimators pointed to a causal effect of higher BMI on an increase in (i) ALT and GGT levels, a novel finding from the present study; (ii) 2-h post-OGTT glucose and fasting insulin; and (iii) diastolic blood pressure and systolic blood pressure. We also observed an unfavorable effect of BMI on lipid metabolism (in individuals without lipid medication), as indicated by decreased levels of HDL-C and increased levels of triglycerides. The IV estimators pointed to a causal link between BMI and inflammation, as indicated by increased levels of CRP. We did not observe a causal effect of BMI on levels of fasting glucose, HbA1c, LDL-C, IL-6, or total cholesterol (Table 3).

Post hoc power calculation showed that for the binary traits with non-significant IVs (CHD, ischemic stroke, and all-cause stroke), we had an 80% chance of detecting an IV estimator odds ratio (OR) of 1.08–1.09/BMI unit or higher, and a 95% chance of detecting an OR of 1.13–1.15/BMI unit or higher. For fasting glucose, we had a 80% chance of detecting a 0.014 mmol/l change per BMI unit and a 95% chance of detecting a 0.022 mmol/l change, smaller than the effect estimate from ordinary linear regression of BMI on glucose (0.028; Table 3).

The causal estimate of the relationship between BMI and ever T2D derived from the MR analysis (the IV estimator) (OR 1.37; 95% CI, 1.23–1.51) was different from the observed association between BMI and ever T2D (OR 1.15; 95% CI, 1.14–1.17; *p* = 0.001).

## Discussion

### Main Findings

In this large-scale meta-analysis, we used a MR design to examine causal associations between adiposity, assessed as elevated BMI, and a number of cardiometabolic outcomes. The present study is, to our knowledge, the most comprehensive MR study published to date, including 24 traits in up to 198,502 individuals with *FTO* genotype and BMI information available. This analysis has enabled us to provide evidence for many biologically plausible causal relationships, such as those between adiposity and hypertension, and between adiposity and dyslipidemia. Furthermore, we demonstrated evidence for a causal relationship between (i) adiposity and heart failure and (ii) adiposity and increased concentrations of the liver enzymes ALT and GGT. In addition, we showed that traditional cross-sectional estimates of the BMI effect on T2D are smaller than the causal estimates of the BMI–T2D relationship based on *FTO*-predicted obesity (IV analyses). This difference is probably driven by lifetime changes in BMI affecting T2D risk, and their attenuation introduced by a single measurement of BMI.

### Comparison with Previous MR Studies

In the present population-based investigation, we confirm earlier findings that *FTO*-mediated adiposity increases the risk of metabolic syndrome and of increased CRP, fasting insulin, and triglyceride levels; increased systolic and diastolic blood pressure; and decreased concentrations of HDL-C [14],[15],[17]–[19].

Using standard regression methods for the association between BMI and other cardiovascular traits, we confirmed associations between adiposity and CHD, ischemic stroke, and all-cause stroke, but did not find an association with hemorrhagic stroke, where we had relatively few cases available for analyses. We could not demonstrate a causal relationship via IV methods applied to these cardiovascular outcomes. The same was true for several metabolic traits, such as for fasting glucose, HbA1c, IL-6, total cholesterol, and LDL-C. However, our findings do not exclude causal relationships as such, since despite the large study sample, the IV analyses brought estimators with rather wide confidence intervals, a common feature when only one genotype is used as an IV. Our calculations showed low power to detect ORs of less than 1.05 in the present study, observed for several BMI–trait associations among those with non-significant IV estimators. We could not find evidence for a causal association between adiposity and all-cause mortality. While the causal association between these phenotypes might be absent, nonlinear relationships, potential survival bias, or low power due to a heterogeneous phenotype could have also affected the results.

We were not able to replicate the findings by Nordestgaard et al., who studied the association between adiposity and CHD using a combined allele score based on *FTO*, *MC4R*, and *TMEM18* variants as an instrument for adiposity, and demonstrated a causal link between BMI and CHD risk [16]. Although the sample sizes and diagnostic criteria were comparable between that study and the present one, Nordestgaard et al. presented more precise estimates, which was probably primarily an effect of the stronger instrument, but the increased precision may also have been influenced by the notion that the ascertainment of CHD events was validated in the three cohorts included, and that results showed low heterogeneity. We found that the IV estimate for the effect of BMI on T2D was higher than that derived from standard logistic regression, which is similar to the finding of Li et al., conducted in east and south Asians [26]. Possible explanations of such an observation include the following: the cross-sectional nature of data that could result in reverse causation (weight loss due to disease or lifestyle interventions), and the notion that the lifelong effect of *FTO* on adiposity is not entirely captured by a single BMI measurement [27].

### Adiposity and Heart Failure

We have provided evidence that the previously suggested association of adiposity with heart failure [28] may indeed be causal. A causal relationship may be mediated through effects of obesity on hypertension, dyslipidemia, and insulin resistance, associations that are also supported by our study. Hypertension, insulin resistance, and T2D have been independently associated with increased risk of heart failure [29],[30]. Hypertension, T2D, dyslipidemia, and insulin resistance are also important risk factors for myocardial infarction, which often results in heart failure [31]. Additionally, increased BMI is associated with cardiac remodeling [32], possibly owing to increased hemodynamic load and increased oxidative stress [33]. Animal models have independently suggested direct apoptotic effects of adiposity on the myocardium [34]. Our study estimates the causal impact of a one-unit increase in BMI as a 17% increase in heart failure incidence. Extrapolating this estimate to the population level based on incidence rates reported by the World Health Organization [35] and the American Heart Association [36], a one-unit increase in BMI corresponds to roughly 220,000 additional heart failure cases in Europe and 113,000 additional cases in the US, at extensive costs for society.

### Adiposity and Liver Enzymes

The higher concentrations of liver enzymes observed in the present study caused by an increased BMI are likely to be related to non-alcoholic fatty liver disease, which is characterized by lipid accumulation within hepatocytes as a consequence of increased levels of fatty acids in insulin-resistant individuals. This accumulation predisposes to overproduction of reactive oxygen species, endoplasmic reticulum stress, and lipotoxicity, all of which are harmful to the hepatocytes [37].

### Strengths and Limitations

The main strengths of the present investigation include the combination of the very large study sample, prospectively collected events, and a wide range of cardiometabolic phenotypes. The limitations of our study are tied to the validity of the assumptions underlying causal interpretation within MR studies. There are three main assumptions for a MR study: (i) independence between the instrument and confounders, i.e., *FTO* genotypes are randomized, (ii) a reliable association between the genetic variant and intermediate phenotype, and (iii) conditional independence between the genetic variant and the outcome, given the intermediate phenotype and the confounders, i.e., no pleiotropy [38]. Possible violations of the first and the third assumptions include population stratification, pleiotropic effects, canalization, epigenetic effects, and the presence of genes associated with confounders and outcomes in LD with the *FTO* variant. Neither the first nor the third assumption can be tested statistically in the observed data using single genotypes as the IV, and conclusions about such assumptions have to be based on previous biological knowledge. There are additional assumptions of MR studies regarding the *quantification* of the causal effect (as opposed to testing only; see Text S1).

The random distribution of genotypes in the population is the very basis of MR and could be violated if separate ethnic groups with different allele frequencies were analyzed together without accounting for the population substructure. In the present study, all association analysis was done within each study (including individuals from a similar genetic background) separately, and all studies included only individuals of European ancestry. Hence, bias from population stratification is deemed unlikely [39].

With regards to the possibility of pleiotropic effects by *FTO* or genes in high LD with *FTO*, we acknowledge that although *FTO* is one of the most well-studied obesity loci, and there are credible hypotheses for its action on adiposity by increasing the appetite [40],[41], the precise mechanism of the *FTO* polymorphisms is still unclear, and potential pleiotropy cannot completely be ruled out. It has, however, been demonstrated previously that *FTO* is not associated with the most obvious potential confounders, such as smoking and drinking habits, income, or education [16]. A suggested way to assess pleiotropy in IV studies using multiple genotypes is to compare IV estimates between variants: if they are similar, it is less plausible that LD or pleiotropy is present [42]. This was done in the study by Nordestgaard and colleagues on the adiposity effect on CHD, and no difference between *FTO*, *MC4R*, and *TMEM18* was seen in effect on CHD risk [16].

Concerning the reliability of the association (second assumption) between rs9939609 and BMI, this association has been widely replicated in many studies and populations [13],[43],[44]. While having the largest effect on BMI among known common variants, *FTO* constitutes a relatively weak instrument and thus results in wide confidence intervals for the IV estimators, despite the very large sample size. An approach to increase power in future studies would be to use multiple genetic variants as an instrument. In the present study, there is a possibility of introduction of a bias by using weak instruments in the calculation of the Wald estimator of dichotomous traits [25]. Our sensitivity analysis (Text S1) estimated that in the settings of our study, the estimator is possibly biased towards the null, and the extent of the bias is modest.

### Conclusion

The present MR study addressing the role of BMI in 24 traits in up to 198,502 individuals provides novel insights into the causal effect of obesity on heart failure and increased liver enzymes levels. Furthermore, to our knowledge for the first time in a well-powered sample, this study provides robust support for a causal relationship between obesity and a number of cardiometabolic traits reported previously. These results support global public prevention efforts for obesity in order to decrease costs and suffering from T2D and heart failure.

## Supporting Information

Figure S1 **Relationship between fixed- and random-effects meta-analysis.**
(DOCX)

Figure S2 **Illustration of sensitivity analysis correlation effect on the instrumental variable estimator for BMI association with ever heart failure.**
(DOCX)

Table S1 **Phenotypic details of the participating cohorts.**
(DOCX)

Table S2 **Cohort-specific genotyping details.**
(DOCX)

Table S3 **List of proxies.**
(DOCX)

Table S4 **Definitions of outcomes and trait transformations.**
(DOCX)

Table S5 **Specifications of assays used for quantitative traits and study-specific definitions of binary traits.**
(DOCX)

Table S6 **Summary of sensitivity analysis for the effect of correlation on instrumental variable estimates.**
(DOCX)

Table S7 **Summary of sensitivity analysis for the effect of correlation on the estimates of differences between instrumental variable and observational analysis.**
(DOCX)

Text S1 **Extended methods.**
(DOCX)

## Abbreviations

ALT: alanine aminotransferase
BMI: body mass index
CRP: C-reactive protein
CHD: coronary heart disease
CVD: cardiovascular disease
GGT: gamma-glutamyl transferase
HbA1c: hemoglobin A1c
HDL-C: high-density-lipoprotein cholesterol
IL-6: interleukin-6
IV: instrumental variable
LD: linkage disequilibrium
LDL-C: low-density lipoprotein cholesterol
MR: Mendelian randomization
OGTT: oral glucose tolerance test
OR: odds ratio
SNP: single nucleotide polymorphism
T2D: type 2 diabetes
