# Supplementary material for: The Role of Adiposity in Cardiometabolic Traits: A Mendelian Randomization Analysis
Source: PLoS Med. 2013 Jun 25;10(6):e1001474. doi: 10.1371/journal.pmed.1001474 (PMC3692470; doi:10.1371/journal.pmed.1001474)
Supplement: Figure S1 — Relationship between fixed- and random-effects meta-analysis. (DOCX) [file pmed.1001474.s001.docx]

**Figure S1.** Distribution of I^2^ (left) and relationship between fixed- and random effect Wald test statistics (right) for IV estimate of the causal effect of BMI on traits (top) and the conventional regression estimate for the effect of BMI on traits (bottom).

Panel A: histogram of I^2^ for the meta-analysis of the effect of *FTO* on each of 30 traits evaluated from more than one study. Panel B: a scatter plot of Wald test statistics for the instrumental variable estimate of BMI on the same 30 traits as in A, with values based on a fixed effect model on the horizontal axis and values based on the random effect model on the vertical axis; each dot corresponds to one trait, with traits “Ever type-2 diabetes”, diastolic blood pressure, fasting glucose, fasting insulin, triglycerides and HDL labelled for illustration. The grey square indicates the area where t-statistics are not significant at the conventional level α=0.05. Panel C: same as A, but for the effect of BMI on the traits. Panel D: same as B, but for the conventional regression estimate of the effect of BMI on traits.
