# Supplementary material for: The Role of Adiposity in Cardiometabolic Traits: A Mendelian Randomization Analysis
Source: PLoS Med. 2013 Jun 25;10(6):e1001474. doi: 10.1371/journal.pmed.1001474 (PMC3692470; doi:10.1371/journal.pmed.1001474)
Supplement: Figure S2 — Illustration of sensitivity analysis correlation effect on the instrumental variable estimator for BMI association with ever heart failure. (DOCX) [file pmed.1001474.s002.docx]

**Figure S2.** Illustration of sensitivity analysis for the instrumental variable estimator of the effect of BMI on a specific trait (here: “Ever heart failure”). On the horizontal axis, the range of possible correlations between $\beta_{FTO\_TRAIT}$ and $\beta_{FTO\_BMI}$ is displayed, on the vertical axis, the effect size (i.e. change of the log-odds ratio of heart failure for a one-point increase of BMI). The thick black horizontal line in the middle represents the reported estimate, the broken horizontal lines at the top and bottom indicate the 95% confidence interval based on the delta method; these do not take the possible correlation into account and are therefore constant in this plot.

The curved thin lines at the top and at the bottom of the plot indicate the 95% confidence interval based on the Fieller theorem, which is clearly wider at the negative end than at the positive end of the correlation scale. The vertical reference lines indicate the width of the confidence intervals for uncorrelated estimates (thin black line at $r=0$) and at the plug-in estimate of the correlation (thick grey line at $r=0.15$). The worst case can clearly be seen at the left margin for $r=-1$.
