# Supplementary material for: The Role of Adiposity in Cardiometabolic Traits: A Mendelian Randomization Analysis
Source: PLoS Med. 2013 Jun 25;10(6):e1001474. doi: 10.1371/journal.pmed.1001474 (PMC3692470; doi:10.1371/journal.pmed.1001474)
Supplement: Table S1 — Phenotypic details of the participating cohorts. (DOCX) [file pmed.1001474.s003.docx]

**Table S1. Phenotypic details of the participating cohorts. (Part 1)**

| **Cohort** | **Full name of cohort** | **Software for analysis** | **Study sample reference** | **Website** |
| --- | --- | --- | --- | --- |
| **DECODE** | deCODE genetics sample set | R | PMID: 18445777, PMID: 17460697,PMID: 18438407, PMID: 19767754, 18991354,17478679 | http://www.decode.com/ |
| **DGIcases** | Diabetes Genetics Initiative of Broad Institute of Harvard and MIT, Lund University, and Novartis Institutes of BioMedical Research | STATA/SE 11.1 | Diabetes Genetics Initiative of Broad Institute of Harvard and MIT, Lund University, and Novartis Institutes of BioMedical Research , et al. Genome-Wide Association Analysis Identifies Loci for Type 2 Diabetes and Triglyceride Levels. Science, 2007 | <http://www.broadinstitute.org/scientific-community/science/projects/diabetes-genetics-initiative/diabetes-genetics-initiative> |
| **DGIcontrols** | Diabetes Genetics Initiative of Broad Institute of Harvard and MIT, Lund University, and Novartis Institutes of BioMedical Research | STATA/SE 11.1 | Diabetes Genetics Initiative of Broad Institute of Harvard and MIT, Lund University, and Novartis Institutes of BioMedical Research, et al. Genome-Wide Association Analysis Identifies Loci for Type 2 Diabetes and Triglyceride Levels. Science 2007 | <http://www.broadinstitute.org/scientific-community/science/projects/diabetes-genetics-initiative/diabetes-genetics-initiative> |
| **DIL** | Wellcome Trust Diabetes and Inflammation Laboratory | Plink and R | Todd, J.A., Walker, N.M., Cooper, J.D., Smyth, D.J. et al (2007) Robust associations of four new chromosome regions from genome-wide analyses of type 1 diabetes. Nat. Genet. | www.wtccc.org.uk / www-gene.cimr.cam.ac.uk |
| **EGCUT** | Estonian Genome Centre of the University of Tartu | R | Nelis et al 2009 PLoS ONE | [www.biobank.ee](http://www.biobank.ee/) |
| **ERF** | Erasmus Rucphen Family (EUROSPAN) | R software | Aulchenko YS, et al. Linkage disequilibrium in young genetically isolated Dutch population. Eur J Hum Genet. 2004 |  |
| **FINNTWIN12** | Finnish Twin cohort / FT12 twins | R 2.12.1 | Kaprio, J et al. Genetic and environmental factors in health-related behaviors: studies on Finnish twins and twin families. Twin Res. 5, 2002 | [www.twinstudy.helsinki.fi](http://www.twinstudy.helsinki.fi/) |
| **FR02** | Finnish Risk factor survey 2002 | R 2.12.1 | Vartiainen E, et al Thirty-five-year trends in cardiovascular risk factors in Finland. Int J Epidemiol. 2010 | www.ktl.fi/finriski |
| **FR07** | Finnish Risk factor survey 2007 | R 2.12.1 | Vartiainen E, et al Thirty-five-year trends in cardiovascular risk factors in Finland. Int J Epidemiol. 2010 | www.ktl.fi/finriski |
| **FR92** | Finnish Risk factor survey 1992 | R 2.12.1 | Vartiainen E,et al . Thirty-five-year trends in cardiovascular risk factors in Finland. Int J Epidemiol. 2010 | www.ktl.fi/finriski |
| **FR97** | Finnish Risk factor survey 1997 | R 2.12.1 | Vartiainen E et al Thirty-five-year trends in cardiovascular risk factors in Finland. Int J Epidemiol. 2010 | www.ktl.fi/finriski |
| **FTC** | FINNISH TWIN COHORT/Nicotine addiction families | Stata 11.2 | Loukola A, et al Linkage of nicotine dependence and smoking behavior on 10q,7q and 11p in twins with homogeneous genetic background. Pharmacogenomics J. 2008 PMID: 17549066. | www.twinstudy.helsinki.fi |
| **GODARTSDIAB** | Genetics of Diabetes Audit and Research Study in Tayside Scotland | SAS/R | PMID:9329309 |  |
| **GODARTSNONDIAB** | Genetics of Diabetes Audit and Research Study in Tayside Scotland | SAS/R | PMID:9329309 |  |
| **GOSH** | Swedish twin registry: Gender, Octo, Satsa, Harmony | STATA 11 | PMID: 17254424 | http://ki.se/ki/jsp/polopoly.jsp?l=en&d=9610 |
| **GRAPHIC** | Genetic Regulation of Arterial Pressure of Humans in the Community | Stata 11 |  |  |
| **H2000** | Finnish Health survey 2000 | R 2.12.1 | Methodology Report - Health 2000 Survey. In: Sami Heistaro, ed. Helsinki: National Public Health Institute; 2008. | www.terveys2000.fi/indexe.html |
| **KORAF3** | Cooperative Health Research in the Region of Augsburg, KOoperative Gesundheitsforschung in der Region Augsburg | R | Wichmann, H.-E.et al (2005): KORA-gen-resource for population genetics, controls and a broad spectrum of disease phenotypes. *Gesundheitswesen* 67 | http://www.helmholtz-muenchen.de/en/kora-en/kora-homepage/index.html |
| **KORAF4** | Cooperative Health Research in the Region of Augsburg, KOoperative Gesundheitsforschung in der Region Augsburg | R | Wichmann, H.-E.et al. (2005): KORA-gen-resource for population genetics, controls and a broad spectrum of disease phenotypes. *Gesundheitswesen* 67 | http://www.helmholtz-muenchen.de/en/kora-en/kora-homepage/index.html |
| **MDCCV** | Malmö Diet and Cancer -cardiovascular cohort (MDC-CV) | SPSS 18 | Hedblad et al: Insulin resistance in non-diabetic subjects is associated with increased incidence of myocardial infarction and death. Diab Med 2002 | www.med.lu.se/klinvetmalmo/mkc_mfm |
| **MORGAM** | Monitoring of trends and determinants in Cardiovascular disease, Risk, Genetics, Archiving and Monograph | R | Evans A, et al , for the MORGAM Project.). Int J Epidemiol 2005;34:21-27. [PubMed], Tunstall-Pedoe H, editor. Prepared by Tunstall-Pedoe et al The WHO MONICA Project. MONICA Monograph and Multimedia Sourcebook. Geneva: World Health Organization; 2003. | http://www.ktl.fi/morgam/ |
| **MPP** | Malmö Prevention Project | STATA/SE 11.1 | Berglund G et al. Cardiovascular risk groups and mortality in an urban Swedish male population: the Malmö Preventive Project. J Intern Med 1996; 239: 489–497 | <http://www.ludc.med.lu.se/research-units/diabetes-and-endocrinology/sample-collections/malmoe-prevention-project-mpp/> |
| **NESDA** | Netherlands Study of Depression and Anxiety | R 2.12.0 / STATA SE 9.0 / PASW 18.0.0 | Penninx, B.W.J.H. et al., 2008, The Netherlands Study of Depression and Anxiety (NESDA): rationale, objectives and methods: Int.J.Methods Psychiatr. Res. | http://www.nesda.nl/en/ |
| **NFBC1966** | Northern Finland Birth Cohort 1966 | SAS 9.2 / R 2.12.2 | Rantakallio P. Groups at risk in low birth weight infants and perinatal mortality. Acta Paediatr Scand. 1969; | [http:kelo.oulu.fi/NFBC/](http://kelo.oulu.fi/NFBC/) |
| **NFBC1986** | Northern Finland Birth Cohort 1986 | SAS 9.2 / R 2.12.2 | Jarvelin MR et al. Labour induction policy in hospitals of different levels of specialisation. Br J Obstet Gynaecol 1993 | [http:kelo.oulu.fi/NFBC/](http://kelo.oulu.fi/NFBC/) |
| **NTR** | Netherlands Twin Register | R 2.12.0 / STATA SE 9.0 / PASW 18.0.0 | Willemsen, G. et al., 2010, The Netherlands Twin Register biobank: a resource for genetic epidemiological studies: Twin Res.Hum.Genet | www.tweelingenregister.org |
| **PIVUS** | Prospective Investigation of the Vasculature in Uppsala Seniors | STATA 11 | E. Ingelsson, J. Hulthe and L. Lind, Inflammatory markers in relation to insulin resistance and the metabolic syndrome, European Journal of Clinical Investigation Vol 38 | http://www.medsci.uu.se/pivus/pivus.htm |
| **PPP** | Prevalence, Prediction and Prevention of diabetes | STATA/SE 11.1 | Isomaa B, Forsén B, Lahti K, et al. A family history of diabetes is associated with reduced physical fitness in the Prevalence, Prediction and Prevention of Diabetes (PPP)–Botnia study. Diabetologia 2010 |  |
| **QIMR-AUSTRALIA** | Twin studies at the Queensland Instutite of Medical Research | Merlin, SPSS | Liu JZ, et al Genome-wide association study of height and body mass index in Australian twin families. Twin Research and Human Genetics 2010 | http://genepi.qimr.edu.au/ |
| **RS** | Rotterdam Study | SPSS | Hofman A, et al . The Rotterdam Study: 2010 objectives and design update. Eur J Epidemiol. 2009 | http://www.epib.nl/research/ergo.htm |
| **TWINGENE** | Cardiovascular risk factor study of Swedish twin pairs | STATA 11 | NA | http://ki.se/ki/jsp/polopoly.jsp?l=en&d=9610 |
| **TwinsUK** | TwinsUK | STATA 10.1 | Spector TD, Williams FM. The UK Adult Twin Registry (TwinsUK). Twin Res Hum Genet. 2006 Dec;9(6):899-906 | http://www.twinsuk.ac.uk/ |
| **ULSAM** | Uppsala longitudinal study of adult men | STATA 11 | Zethelius B, et al Proinsulin and acute insulin response independently predict Type 2 diabetes mellitus in men--report from 27 years of follow-up study. Diabetologia 2003 | <http://www.pubcare.uu.se/ULSAM> |
| **WTCCCCases** | Wellcome Trust Case Control Consortium CAD Cases | Stata 11 & Plink | WTCCC2 Nature. 2010;464;713-20. PMID: 20360734 DOI: 10.1038/nature08979 | www.wtccc.org.uk |
| **WTCCCCont** | Wellcome Trust Case Control Consortium 1958 Birth Cohort | Stata 11 & Plink | WTCCC -58BC .C. Power, J. Elliott, Int. J. Epidemiol., 35, 34 (2006). | www.wtccc.org.uk |
| **WTCCCT2D** | Wellcome Trust Case Control Consortium - T2D | PLINK / R | WTCCC-T2D, Diabetes UK Warren 2 repository S. Wiltshire et al., Am. J. of Hum. Gen. 69, 553-569 (2001). | www.wtccc.org.uk |

**TableS1**. **Phenotypic details of the participating cohorts**. **(Part 2)**

|  |  | |  |  | |  | |  | |  | | |  | | |  |
| --- | --- | --- | --- | --- | --- | --- | --- | --- | --- | --- | --- | --- | --- | --- | --- | --- |
| **Cohort** | | **Number of subjects with *FTO* and BMI data** | | | **Longitudinal data** | | **Age at BMI** | | **Mean BMI at baseline** | | **Proportion women (%)** | **N with incident type 2 diabetes** | | **N with ever type 2 diabetes** | **N with incident any stroke** | **N with ever any stroke** |
| **DECODE** | | 36,896 | | | No | | 59.1 (18.0) | | 27.2 (5.3) | | 63.8% | NA | | 2,126 | NA | NA |
| **DGIcases** | | 1602 | | | No | | 64.42 (10.32) | | 28.50 (4.40) | | 49.8% | NA | | NA | NA | 201 |
| **DGIcontrols** | | 1508 | | | No | | 58.61 (10.16) | | 26.70 (3.78) | | 52.2% | NA | | NA | NA | 26 |
| **DIL** | | 2,589 | | | No | | 45 (0) | | 27.5 (5.0) | | 51.3% | NA | | NA | NA | NA |
| **EGCUT** | | 11,282 | | | No | | 45.74 (18.34) | | 26.5 (5.52) | | 56.0% | NA | | 1,132 | NA | 323 |
| **ERF** | | 2,725 | | | No | | 48.9(14.3) | | 26.9 (4.7) | | 55.3% | NA | | 146 | NA | NA |
| **FINNTWIN12** | | 419 | | | No | | 22.9 (1.5) | | 22.84 (4.0) | | 49.2% | NA | | NA | NA | NA |
| **FR02** | | 8,142 | | | Yes | | 48.9(13.12) | | 26.91 (4.68) | | 53.3% | 296 | | 746 | 96 | 228 |
| **FR07** | | 5,900 | | | Yes | | 50.45 (13.93) | | 27.13 (4.88) | | 53.3% | 67 | | 567 | 18 | 121 |
| **FR92** | | 5,536 | | | Yes | | 44.39 (11,32) | | 26.13 (4.46) | | 53.9% | 426 | | 629 | 194 | 253 |
| **FR97** | | 6,747 | | | Yes | | 47.79 (13.22) | | 26.63 (4.61) | | 53.3% | 429 | | 818 | 188 | 303 |
| **FTC** | | 1,109 | | | Yes | | 55.01 (4.4.3) | | 26.09 (4.07) | | 38.1% | NA | | 55 | NA | NA |
| **GODARTSDIAB** | | 8,171 | | | No | | 66.11(11.28) | | 31.37(6.12) | | 44.0% | NA | | NA | NA | 629 |
| **GODARTSNONDIAB** | | 6,768 | | | No | | 60.73(13.09) | | 27.18(4.60) | | 49.9% | NA | | 8,178 | NA | 202 |
| **GOSH** | | 1,346 | | | Yes | | 54.17(12.00) | | 24.52(3.20) | | 50.1% | NA | | 62 | 289 | 316 |
| **GRAPHIC** | | 2,024 | | | No | | 39.29 (14.5) | | 26.11 (4.6) | | 49.6% | NA | | 21 | NA | NA |
| **H2000** | | 3,480 | | | Yes | | 56.21 (17.19) | | 26.81 (4.68) | | 56.9% | NA | | 311 | 93 | 261 |
| **KORAF3** | | 2,976 | | | No | | 56.92 (12.76) | | 27.61 (4.62) | | 52.3% | NA | | 238 | NA | NA |
| **KORAF4** | | 3,009 | | | No | | 56.08 (13.26) | | 27.62 (4.81) | | 51.5% | NA | | 214 | NA | NA |
| **MDCCV** | | 5,901 | | | No | | 57.47 (5.94) | | 25.77 (3.99) | | 58.0% | NA | | 420 | NA | NA |
| **MORGAM** | | 3,745 | | | Yes | | 59.28 (6.93) | | 26.75 (3.99) | | 4.6% | NA | | 267 | 356 | 139 |
| **MPP** | | 13,616 | | | No | | 45.2 (7.01) | | 24.28 (3.30) | | 33.3% | NA | | NA | NA | NA |
| **NESDA** | | 1,927 | | | No | | 41.90 (12.52) | | 25.65 (5.04) | | 67.6% | NA | | 95 | NA | NA |
| **NFBC1966** | | 4,775 | | | Yes | | 31.17 (0.35) | | 24.70 (4.28) | | 51.8% | NA | | 123 | 20 | 33 |
| **NFBC1986** | | 5,285 | | | Yes | | 16.00 (0.37) | | 21.22 (3.48) | | 51.0% | NA | | NA | NA | NA |
| **NTR** | | 5,416 | | | No | | 42.55 (14.76) | | 25.25 (4.30) | | 61.2% | NA | | 240 | NA | NA |
| **PIVUS** | | 979 | | | No | | 70.19 (0.17) | | 27.07 (4.3) | | 49.8% | NA | | 34 | NA | 35 |
| **PPP** | | 4,355 | | | No | | 47.90 (15.63) | | 26.31 (4.44) | | 53.8% | NA | | 160 | NA | 49 |
| **QIMR-AUSTRALIA** | | 11,827 | | | No | | 35.61 (17.41) | | 24.12 (5.12) | | 57.2% | NA | | NA | NA | NA |
| **RS** | | 5,745 | | | Yes | | 69.0 (8.80) | | 26.3 (3.69) | | 58.7% | 547 | | 1,178 | 618 | 149 |
| **TWINGENE** | | 6,386 | | | Yes | | 65.4(8.3) | | 26.2(4.2) | | 45.0% | NA | | 640 | 327 | 461 |
| **TwinsUK** | | 4,829 | | | No | | 52.8(14.42) | | 26.1 (5.06) | | 0.0% | NA | | 80 | NA | NA |
| **ULSAM** | | 1,175 | | | Yes | | 49.6 (0.6) | | 24.8 (3.0) | | 0.0% | 226 | | 48 | 274 | 274 |
| **WTCCCCases** | | 2,966 | | | No | | 51.27 (8.7) | | 28.02 (4.5) | | 19.9% | NA | | 260 | NA | NA |
| **WTCCCCont** | | 5,443 | | | No | | 46(0) | | 27.37 (4.8) | | 45.7% | NA | | 113 | NA | NA |
| **WTCCCT2D** | | 1,903 | | | No | | 48.71 (10.41) | | 28.23 (5.57) | | 47.4% | NA | | 1,903 | NA | NA |

**Table S1**. **Phenotypic details of the participating cohorts**. **(Part 3)**

| **Cohort** | **N with mortality events** | **N with incident metabolic syndrome** | **N with ever metabolic syndrome** | **N with incident ischemic stroke** | **N with ever ischemic stroke** | **N with incident hypertension** | **N with ever hypertension** | **N with incident hemorrhagic stroke** | **N with ever hemorrhagic stroke** |
| --- | --- | --- | --- | --- | --- | --- | --- | --- | --- |
| **DECODE** | NA | NA | NA | NA | 2,366 | NA | 8,248 | NA | 284 |
| **DGIcases** | NA | NA | 1,008 | NA | NA | NA | 1,101 | NA | NA |
| **DGIcontrols** | NA | NA | 353 | NA | NA | NA | 666 | NA | NA |
| **DIL** | NA | NA | NA | NA | NA | NA | NA | NA | NA |
| **EGCUT** | 281 | NA | 1,101 | NA | 128 | NA | 4,362 | NA | 10 |
| **ERF** | NA | NA | 403 | NA | NA | NA | 1,285 | NA | NA |
| **FINNTWIN12** | NA | NA | NA | NA | NA | NA | NA | NA | NA |
| **FR02** | 253 | NA | NA | 69 | 157 | NA | 3,569 | NA | NA |
| **FR07** | 36 | NA | 1,596 | 11 | 83 | NA | 2,874 | NA | NA |
| **FR92** | 493 | NA | NA | 140 | 175 | NA | 2,415 | NA | NA |
| **FR97** | 565 | NA | NA | 154 | 235 | NA | 3,191 | NA | NA |
| **FTC** | 57 | NA | NA | NA | NA | NA | NA | NA | NA |
| **GODARTSDIAB** | NA | NA | NA | NA | 181 | NA | NA | NA | 67 |
| **GODARTSNONDIAB** | NA | NA | NA | NA | 37 | NA | NA | NA | 41 |
| **GOSH** | 959 | NA | NA | 190 | 212 | NA | 1,189 | 55 | 56 |
| **GRAPHIC** | NA | NA | NA | NA | NA | NA | 577 | NA | NA |
| **H2000** | 558 | NA | NA | 75 | 225 | NA | 1,828 | NA | NA |
| **KORAF3** | NA | NA | NA | NA | NA | NA | 1,476 | NA | NA |
| **KORAF4** | NA | NA | 583 | NA | NA | NA | 1,158 | NA | NA |
| **MDCCV** | NA | NA | 1,450 | NA | NA | NA | 3,049 | NA | NA |
| **MORGAM** | 1,434 | NA | NA | 282 | NA | NA | 1,966 | 64 | NA |
| **MPP** | NA | NA | 24 | NA | NA | NA | 4,700 | NA | NA |
| **NESDA** | NA | NA | NA | NA | NA | NA | NA | NA | NA |
| **NFBC1966** | 49 | NA | 664 | 8 | 13 | NA | 419 | 6 | 11 |
| **NFBC1986** | 21 | NA | 110 | NA | NA | NA | 17 | NA | NA |
| **NTR** | NA | NA | NA | NA | NA | NA | NA | NA | NA |
| **PIVUS** | NA | NA | 120 | NA | NA | NA | 144 | NA | NA |
| **PPP** | NA | NA | 1,034 | NA | NA | NA | 1,778 | NA | NA |
| **QIMR-AUSTRALIA** | NA | NA | NA | NA | NA | NA | NA | NA | NA |
| **RS** | 2,980 | NA | 1,291 | 340 | NA | NA | 3,273 | 56 | NA |
| **TWINGENE** | 276 | NA | 1,766 | 181 | 254 | NA | 3,771 | 48 | 68 |
| **TwinsUK** | NA | NA | 80 | NA | NA | NA | 572 | NA | NA |
| **ULSAM** | 735 | 458 | 76 | 167 | 167 | 600 | 438 | 51 | 51 |
| **WTCCCCases** | NA | NA | NA | NA | NA | NA | 1,228 | NA | NA |
| **WTCCCCont** | NA | NA | NA | NA | NA | NA | 1,427 | NA | NA |
| **WTCCCT2D** | NA | NA | NA | NA | NA | NA | NA | NA | NA |

**Table S1**. **Phenotypic details of the participating cohorts**. **(Part 4)**

| **Cohort** | **N with incident heart failure** | **N with ever heart failure** | **N with incident dyslipidemia** | **N with ever dyslipidemia** | **N with incident coronary heart disease** | **N with ever coronary heart disease** | **N with total cholesterol** | **N with systolic blood pressure** | **N with triglycerites** |
| --- | --- | --- | --- | --- | --- | --- | --- | --- | --- |
| **DECODE** | NA | NA | NA | NA | NA | 3568 | 18,393 | 16,726 | 17,099 |
| **DGIcases** | NA | NA | NA | 1,038 | NA | 447 | 1,455 | 1,584 | 1,455 |
| **DGIcontrols** | NA | NA | NA | 616 | NA | 36 | 1,416 | 1,503 | 1,416 |
| **DIL** | NA | NA | NA | NA | NA | NA | 2,526 | 2,577 | 2,514 |
| **EGCUT** | NA | 804 | NA | 3,268 | NA | 537 | 2,362 | 11,277 | 1,868 |
| **ERF** | NA | NA | NA | 333 | NA | NA | 2,716 | 2,728 | 2,713 |
| **FINNTWIN12** | NA | NA | NA | NA | NA | NA | 416 | NA | 424 |
| **FR02** | 231 | 506 | NA | 3,074 | 162 | 383 | 7,549 | 8,142 | 7,549 |
| **FR07** | 65 | 284 | NA | 1,919 | 33 | 196 | 5,066 | 5,877 | 3,991 |
| **FR92** | 513 | 605 | NA | 2,142 | 331 | 410 | 5,451 | 5,537 | 5,450 |
| **FR97** | 467 | 711 | NA | 2,733 | 347 | 516 | 6,594 | 6,807 | 6,594 |
| **FTC** | NA | NA | NA | NA | NA | NA | NA | NA | NA |
| **GODARTSDIAB** | NA | 747 | NA | NA | NA | 1,030 | 7,679 | 8,077 | 5,724 |
| **GODARTSNONDIAB** | NA | 139 | NA | NA | NA | 262 | 6,407 | 6,760 | 5,529 |
| **GOSH** | 223 | 240 | NA | 477 | 96 | 102 | 1,374 | 1,344 | 1,102 |
| **GRAPHIC** | NA | NA | NA | NA | NA | NA | 2,020 | 2,020 | 1,961 |
| **H2000** | 308 | 745 | NA | 1,547 | 175 | 437 | 2,846 | 3,244 | 2,846 |
| **KORAF3** | NA | NA | NA | 397 | NA | NA | 231 | 2,985 | 231 |
| **KORAF4** | NA | NA | NA | 303 | NA | NA | 3,008 | 3,018 | 3,007 |
| **MDCCV** | NA | NA | NA | 2,089 | NA | NA | 5,210 | 5,901 | 5,210 |
| **MORGAM** | NA | NA | NA | 1,565 | 973 | 341 | 3,628 | 3,754 | 1,714 |
| **MPP** | NA | NA | NA | NA | NA | NA | 10,880 | 9,853 | 10,870 |
| **NESDA** | NA | NA | NA | 544 | NA | NA | 1,813 | NA | 1,810 |
| **NFBC1966** | 4 | 8 | NA | 1,031 | 14 | 17 | 4,566 | 4,769 | 4,565 |
| **NFBC1986** | NA | NA | NA | 1,030 | NA | NA | 5,110 | 5,281 | 5,110 |
| **NTR** | NA | NA | NA | 2,043 | NA | NA | 5,032 | NA | 5,032 |
| **PIVUS** | NA | NA | NA | 90 | NA | 27 | 784 | 975 | 784 |
| **PPP** | NA | NA | NA | 1,734 | NA | 210 | 3,923 | 4,355 | 3,924 |
| **QIMR-AUSTRALIA** | NA | NA | NA | NA | NA | NA | 8,315 | NA | 8,311 |
| **RS** | 717 | 894 | NA | 1,313 | 1056 | 1557 | 3,382 | 5,791 | 3,230 |
| **TWINGENE** | 142 | 174 | NA | 2,820 | 24 | 25 | 5,401 | 6,101 | 5,398 |
| **TwinsUK** | NA | NA | NA | 747 | NA | NA | 4,245 | 2,646 | 4,194 |
| **ULSAM** | 211 | 211 | 237 | 561 | 271 | 271 | 1,128 | 1,175 | 1,128 |
| **WTCCCCases** | NA | NA | NA | NA | NA | NA | 1,341 | 1,407 | 1,147 |
| **WTCCCCont** | NA | NA | NA | NA | NA | NA | 5,352 | 5,430 | 5,341 |
| **WTCCCT2D** | NA | NA | NA | NA | NA | NA | NA | NA | NA |

**Table S1**. **Phenotypic details of the participating cohorts**. **(Part 5)**

| **Cohort** | **N with interleukine 6** | **N with gamma-glytamyl-transferase** | **N with fasting insulin** | **N with c-reactive protein** | **N with C-peptide** | **N with alanine aminotransferase** | **N with LDL cholesterol** | **N with HDL cholesterol** |
| --- | --- | --- | --- | --- | --- | --- | --- | --- |
| **DECODE** | NA | NA | 3,103 | 24,128 | NA | NA | 16,297 | 17,009 |
| **DGIcases** | NA | NA | NA | NA | NA | NA | 426 | 1,414 |
| **DGIcontrols** | NA | NA | 1,345 | NA | NA | NA | 710 | 1,406 |
| **DIL** | NA | NA | NA | 2,475 | NA | NA | 2,373 | 2,519 |
| **EGCUT** | NA | 859 | NA | NA | NA | 859 | 1,872 | 1,921 |
| **ERF** | NA | NA | 2,125 | 2,090 | NA | NA | 2,699 | 2,716 |
| **FINNTWIN12** | NA | NA | NA | NA | NA | NA | 424 | 424 |
| **FR02** | NA | 8,138 | NA | 8,119 | NA | NA | 7,454 | 7,549 |
| **FR07** | NA | 5,900 | 3,865 | 5,900 | NA | NA | 3,990 | 3,991 |
| **FR92** | NA | 5,524 | NA | 932 | NA | NA | 5,330 | 5,451 |
| **FR97** | NA | 6,811 | NA | 6,457 | NA | NA | 6,480 | 6,594 |
| **FTC** | NA | NA | NA | NA | NA | NA | NA | NA |
| **GODARTSDIAB** | NA | NA | NA | NA | NA | NA | 5,172 | 7,343 |
| **GODARTSNONDIAB** | NA | NA | 1,642 | NA | NA | NA | 5,365 | 6,307 |
| **GOSH** | NA | 310 | 109 | 282 | NA | NA | 856 | 1,166 |
| **GRAPHIC** | NA | NA | NA | NA | NA | NA | 1,961 | 2,020 |
| **H2000** | NA | NA | 2,577 | NA | NA | NA | 2,831 | 2,846 |
| **KORAF3** | 1,537 | 2,996 | NA | 243 | NA | 2,994 | 231 | 231 |
| **KORAF4** | NA | 3,017 | 2,981 | 3,018 | NA | 3,017 | 3,007 | 3,007 |
| **MDCCV** | NA | NA | 4,755 | NA | NA | NA | 5,077 | 5,156 |
| **MORGAM** | NA | NA | NA | NA | NA | NA | 1,663 | 3,628 |
| **MPP** | NA | 13,563 | NA | NA | NA | 12,318 | NA | 243 |
| **NESDA** | 1,905 | 1,877 | NA | 1,901 | NA | NA | 1,795 | 1,806 |
| **NFBC1966** | NA | NA | 4,297 | 4,755 | NA | NA | 4,551 | 4,566 |
| **NFBC1986** | NA | NA | 4,879 | 5,247 | NA | NA | 5,110 | 5,110 |
| **NTR** | 4,804 | 5,052 | 4,707 | 4,958 | NA | 4,855 | 5,021 | 5,030 |
| **PIVUS** | 961 | 975 | 892 | 972 | NA | 975 | 782 | 784 |
| **PPP** | NA | NA | 4,142 | NA | 1,195 | 4,333 | 3,881 | 3,923 |
| **QIMR-AUSTRALIA** | NA | 8,241 | NA | NA | NA | 8,242 | 7,962 | 8,278 |
| **RS** | 599 | 4,309 | 3,414 | 5,567 | NA | 4,314 | 3,140 | 3,331 |
| **TWINGENE** | NA | NA | NA | 6,489 | NA | NA | 5,322 | 5,401 |
| **TwinsUK** | 398 | 3,546 | 2,272 | 4,035 | NA | 3,761 | 4,183 | 4,247 |
| **ULSAM** | 1,021 | NA | 913 | 1,082 | NA | 1,086 | 917 | 917 |
| **WTCCCCases** | NA | NA | NA | NA | NA | NA | 1,103 | 1,103 |
| **WTCCCCont** | NA | NA | NA | 2,687 | NA | NA | 5,041 | 5,345 |
| **WTCCCT2D** | NA | NA | NA | NA | NA | NA | NA | NA |

**Table S1. Phenotypic details of the participating cohorts. (Part 6)**

| **Cohort** | **N with HbA1c** | **N with fasting glucose** | **N with diastolic blood pressure** | **N with 2h post OGTT glucose** | **Glucose: mean (SD) / mmol/L** | **Insulin: mean (SD) /pmol/L** | **HbA1c: mean (SD)/%** | **2hG: mean (SD) / mmol/L** | **C-peptide: mean (SD) / nmol/l** | **HDL-C: mean (SD) / mmol/L** | **LDL-C: mean (SD) / mmol/L** |
| --- | --- | --- | --- | --- | --- | --- | --- | --- | --- | --- | --- |
| **DECODE** | NA | 12,017 | NA | NA | 5.25 (0.60) | 63.6 (55.1) | NA | NA | NA | 1.45 (0.42) | 3.69 (1.08) |
| **DGIcases** | NA | NA | 1,583 | NA | NA | NA | NA | NA | NA | 1.20 (0.31) | 3.96 (1.04) |
| **DGIcontrols** | 505 | 1,387 | 1,502 | 1,017 | 5.32 (0.52) | 39.47 (32.41) | 5.37 (0.52) | 5.64 (1.32) | NA | 1.39 (0.34) | 4.03 (0.92) |
| **DIL** | 2,532 | NA | 2,577 | NA | NA | NA | 5.21 (0.64) | NA | NA | 1.56 (0.40) | 3.41 (0.91) |
| **EGCUT** | NA | 1,757 | 11,277 | 301 | 5.38 (0.79) | NA | NA | 5.92 (1.20) | NA | 1.36 (0.70) | 3.60 (1.63) |
| **ERF** | NA | 2,703 | 2,728 | NA | 4.66 (1.15) | 13.26 (7.51) | NA | NA | NA | 1.28 (0.37) | 3.72 (0.98) |
| **FINNTWIN12** | NA | NA | NA | NA | NA | NA | NA | NA | NA | 1.76 (0.41) | 1.68 (0.47) |
| **FR02** | NA | NA | 8,142 | NA | NA | NA | NA | NA | NA | 1.51 (0.42) | 3.45 (0.95) |
| **FR07** | NA | 3,872 | 5,874 | 3,827 | 5.72 (0.46) | 42.42 (25.31) | NA | 6.16 (1.65) | NA | 1.46 (0.35) | 3.28 (0.82) |
| **FR92** | NA | NA | 5,536 | NA | NA | NA | NA | NA | NA | 1.40 (0.35) | 3.55 (0.99) |
| **FR97** | NA | NA | 6,808 | NA | NA | NA | NA | NA | NA | 1.40 (0.36) | 3.48 (0.92) |
| **FTC** | NA | NA | NA | NA | NA | NA | NA | NA | NA | NA | NA |
| **GODARTSDIAB** | NA | NA | 8,077 | NA | 9.58 (3.55) | NA | 7.20(1.44) | NA | NA | 1.26 (0.67) | 2.71 (1.01) |
| **GODARTSNONDIAB** | 6,728 | 3,388 | 6,760 | NA | 5.13 (0.91) | 78.06 (60.00) | 5.52 (0.42) | NA | NA | 1.59 (0.57) | 3.10 (0.97) |
| **GOSH** | 375 | 102 | 1,344 | NA | 6.3(0.55) | 63.5(34.1) | 5.51(1.00) | NA | NA | 1.43(0.41) | 3.75(1.00) |
| **GRAPHIC** | NA | NA | 2,020 | NA | NA | NA | NA | NA | NA | 1.63 (0.4) | 2.89 (0.8) |
| **H2000** | NA | 2,660 | 3,244 | NA | 5.94 (0.46) | 53.62 (36.55) | NA | NA | NA | 1.33 (0.38) | 3.74 (1.06) |
| **KORAF3** | 2,999 | 231 | 2,985 | NA | 6.41 (1.18) | NA | 5.36 (0.52) | NA | NA | 1.51 (0.46) | 3.48 (0.91) |
| **KORAF4** | 3,024 | 2,990 | 3,018 | 2,724 | 6.17 (1.20) | 65.09 (249.2) | 5.55 (0.62) | 7.06 (2.47) | NA | 1.44 (0.37) | 3.51 (0.90) |
| **MDCCV** | 4,895 | 4,914 | 5,901 | NA | 5.85 (1.56) | 47.93 (45.22) | 4.91 (0.77) | NA | NA | 1.38 (0.37) | 4.17 (0.99) |
| **MORGAM** | NA | NA | 3,754 | NA | NA | NA | NA | NA | NA | 1.21 (0.39) | 3.81 (0.95) |
| **MPP** | 202 | 13,615 | 9,858 | 7,370 | 5.46 (0.554) | NA | 4.83 (0.49) | 5.64 (1.47) | NA | 1.55 (0.37) | NA |
| **NESDA** | NA | 1,722 | NA | NA | 5.03 (0.58) | NA | NA | NA | NA | 1.63 (0.44) | 3.13 (1.00) |
| **NFBC1966** | NA | 4,322 | 4,762 | NA | 5.70 (0.63) | 58.47 (28.71) | NA | NA | NA | 1.55 (0.38) | 3.00 (0.89) |
| **NFBC1986** | NA | 4,789 | 5,281 | NA | 5.18 (0.73) | 76.10 (49.32) | NA | NA | NA | 1.40 (0.29) | 2.25 (0.57) |
| **NTR** | 4,725 | 4,821 | NA | NA | 5.35 (0.53) | 63.03 (42.14) | 5.31 (0.57) | NA | NA | 1.42 (0.38) | 3.10 (0.95) |
| **PIVUS** | NA | 855 | 975 | NA | 5.57 (0.56) | 51.0 (30.6) | NA | NA | NA | 1.52 (0.43) | 3.40 (0.84) |
| **PPP** | NA | 4,173 | 4,355 | 4,144 | 5.28 (0.57) | 45.83 (34.66) | NA | 5.24 (1.59) | 0.64 (0.26) | 1.42 (0.39) | 3.30 (0.944) |
| **QIMR-AUSTRALIA** | NA | NA | NA | NA | NA | NA | NA | NA | NA | 1.52 (0.42) | 3.31 (0.93) |
| **RS** | NA | 3,295 | 5,790 | NA | 5.88 (1.32) | 96.1 (253.1) | NA | NA | NA | 1.34 (0.37) | 3.75 (0.88) |
| **TWINGENE** | 6,484 | 5,657 | 6,044 | NA | 5.40 (0.60) | NA | 4.80 (0.07) | NA | NA | 1.4(0.41) | 3.90 (0.93) |
| **TwinsUK** | NA | 4,517 | 2,646 | 966 | 4.66 (0.58) | 49.53 (34.40) | NA | 6.78 (0.40) | NA | 1.47 (0.40) | 3.41 (1.09) |
| **ULSAM** | 288 | 1,123 | 1,175 | 908 | 5.50 (0.50) | 73.40 (39.70) | 7.10 (0.99) | 6.90 (1.80) | NA | 1.40 (0.40) | 5.20 (1.20) |
| **WTCCCCases** | NA | NA | 934 | NA | NA | NA | NA | NA | NA | 1.11 (0.40) | 2.95 (0.90) |
| **WTCCCCont** | 2,714 | NA | 5,430 | NA | NA | NA | 5.27 (0.69) | NA | NA | 1.11 (0.40) | 2.95 (0.90) |
| **WTCCCT2D** | NA | NA | NA | NA | NA | NA | NA | NA | NA | NA | NA |

**Table S1**. **Phenotypic details of the participating cohorts**. **(Part 7)**

|  |  | |  | |  |  | | |  | |  |  |
| --- | --- | --- | --- | --- | --- | --- | --- | --- | --- | --- | --- | --- |
| **Cohort** | **Total cholesterol: mean (SD) / mmol/L** | **Systolic blood pressure: mean (SD) / mmHg** | | **Diastolic blood pressure: mean (SD) / mmHg** | | | **ALT: mean (SD) / U/L** | **GGT: mean (SD) / U/L** | | **Interleukine-6: mean (SD) / pg/mL** | | **CRP: mean (SD) / mg/L** |
| **DECODE** | 5.82 (1.17) | 135.4 (20.4) | | NA | | | NA | NA | | NA | | 38.7 (65.8) |
| **DGIcases** | 5.81 (1.18) | 149.2 (20.8) | | 84.1 (10.2) | | | NA | NA | | NA | | NA |
| **DGIcontrols** | 5.93 (1.09) | 135.9 (18.8) | | 81.5 (9.9) | | | NA | NA | | NA | | NA |
| **DIL** | 5.88 (1.10) | 126.8 (16.4) | | 79.0 (10.8) | | | NA | NA | | NA | | 2.12 (3.80) |
| **EGCUT** | 5.50 (1.18) | 130.81 (21.75) | | 80.52 (12.92) | | | 24.3 (17.3) | 21.5 (20.3) | | NA | | NA |
| **ERF** | 5.56 (1.10) | 140.30 (20.44) | | 80.46 (10.03) | | | NA | NA | | NA | | 3.36 (8.12) |
| **FINNTWIN12** | 4.64 (0.82) | NA | | NA | | | NA | NA | | NA | | NA |
| **FR02** | 5.60 (1.07) | 137.13 (22.02) | | 80.46 (12.53) | | | NA | 34.67 (51.55) | | NA | | 2.49 (5.23) |
| **FR07** | 5.32 (0.99) | 138.92 (22.69) | | 81.01 (12.55) | | | NA | 33.36 (47.18) | | NA | | 2.43 (5.03) |
| **FR92** | 5.62 (1.11) | 136.84 (20.98) | | 82.18 (12.93) | | | NA | 27.64 (40.77) | | NA | | 4.05 (7.63) |
| **FR97** | 5.54 (1.05) | 137.63 (21.78) | | 83.52 (12.47) | | | NA | 35.23 (53.19) | | NA | | 2.38 (5.91) |
| **FTC** | NA | NA | | NA | | | NA | NA | | NA | | NA |
| **GODARTSDIAB** | 5.66 (1.27) | 141.50(18.55) | | 76.28 (11.09) | | | NA | NA | | NA | | NA |
| **GODARTSNONDIAB** | 5.58 (1.04) | 135.90 (19.36) | | 79.28 (9.95) | | | NA | NA | | NA | | NA |
| **GOSH** | 6.3(1.35) | 154.3(25.1) | | 84.3(12.5) | | | NA | 0.53(0.57) | | NA | | 7.23(5.8) |
| **GRAPHIC** | 5.08 (1.1) | 119.47 (11.4) | | 72.15 (8.1) | | | NA | NA | | NA | | NA |
| **H2000** | 5.96 (1.13) | 140.47 (25.21) | | 84.79 (12.97) | | | NA | NA | | NA | | NA |
| **KORAF3** | 5.81 (1.02) | 135.21 (22.48) | | 84.95 (11.61) | | | 20.78 (12.57) | 38.2 (58.01) | | 2.85 (10.45) | | 0.45 (0.85) |
| **KORAF4** | 5.58 (1.02) | 126.94 (21.19) | | 78.25 (10.95) | | | 25.69 (12.24) | 40.4 (77.23) | | NA | | 0.25 (0.53) |
| **MDCCV** | 6.17 (1.10) | 141.33 (19.04) | | 86.98 (9.47) | | | NA | NA | | NA | | NA |
| **MORGAM** | 5.92 (1.06) | 142 (21) | | 86 (12) | | | NA | NA | | NA | | NA |
| **MPP** | 5.61 (1.04) | 127.1 (14.2) | | 83.9 (8.8) | | | 0.367 (0.210) | 0.497 (0.410) | | NA | | NA |
| **NESDA** | 5.11 (1.06) | NA | | NA | | | NA | 25.50 (29.47) | | 1.64 (16.21) | | 2.84 (5.13) |
| **NFBC1966** | 5.06 (0.99) | 125.21 (13.88) | | 77.69 (11.60) | | | NA | NA | | NA | | 2.01 (3.66) |
| **NFBC1986** | 4.26 (0.79) | 115.48 (12.73) | | 67.69 (7.58) | | | NA | NA | | NA | | 0.99 (2.85) |
| **NTR** | 5.12 (1.06) | NA | | NA | | | 10.33 (6.14) | 32.02 (34.39) | | 1.90 (4.31) | | 3.30 (6.54) |
| **PIVUS** | 5.4 (0.98) | 149.7 (22.7) | | 78.8 (10.2) | | | 20.2 (13.5) | 29.9 (30.0) | | 30.4 (85.3) | | 3.2 (4.8) |
| **PPP** | 5.30 (1.06) | 129.3 (17.2) | | 79.1 (9.9) | | | 27.9 (19.9) | NA | | NA | | NA |
| **QIMR-AUSTRALIA** | 5.67 (1.05) | NA | | NA | | | 24.46 (17.32) | 29.32 (35.69) | | NA | | NA |
| **RS** | 6.59 (1.22) | 139.2 (22.3) | | 73.7 (11.5) | | | 18.0 (11.4) | 29.9 (28.6) | | 2.827 (4.03) | | 3.38 (6.8) |
| **TWINGENE** | 5.94(1.1) | 139.3(19.8) | | 81.6(10.5) | | | NA | NA | | NA | | 3.42(7.4) |
| **TwinsUK** | 5.44 (1.23) | 121.41 (15.91) | | 76.60 (10.56) | | | 28.55 (13.68) | 27.04 (24.70) | | 23.29 (26.59) | | 2.65 (4.70) |
| **ULSAM** | 6.8 (1.3) | 131.4 (16.8) | | 82.6 (10.5) | | | 25.8(15.3) | NA | | 5.85(9.2) | | 3.3 (4.7) |
| **WTCCCCases** | 5.00 (1.1) | 139.46 (24.0) | | 81.14 (13.4) | | | NA | NA | | NA | | NA |
| **WTCCCCont** | 5.00 (1.1) | 139.46 (24.0) | | 81.14 (13.4) | | | NA | NA | | NA | | 2.20 (4.32) |
| **WTCCCT2D** | NA | NA | | NA | | | NA | NA | | NA | | NA |
