# Supplementary material for: The Role of Adiposity in Cardiometabolic Traits: A Mendelian Randomization Analysis
Source: PLoS Med. 2013 Jun 25;10(6):e1001474. doi: 10.1371/journal.pmed.1001474 (PMC3692470; doi:10.1371/journal.pmed.1001474)
Supplement: Table S2 — Cohort-specific genotyping details. (DOCX) [file pmed.1001474.s004.docx]

**Table S2. Cohort-specific genotyping details**

|  | **SNP** | **EFFECT ALLELE** | **NON EFFECT ALLELE** | **EAF** | **HWE  p-value** | **Call rate** | **IMP** | **In silico/**  **De novo** | **Genotyping platform & SNP panel** | **Genotyping centre** | **Genotyping calling algorithm** |
| --- | --- | --- | --- | --- | --- | --- | --- | --- | --- | --- | --- |
| **DECODE** | rs9939609 | A | T | 0.41 | 0.54 | . | 0 | De novo/  In silico | Centaurus (Nanogen) | DeCode | - |
| **DGIcases** | rs9939609 | A | T | 0.42 | 0.64 | 1 | 0 | In silico | Affymetrix GeneChip® Human Mapping 500K Array Set | Broad Institute | BRLMM |
| **DGIcontrols** | rs9939609 | A | T | 0.40 | 0.24 | 1 | 0 | In silico | Affymetrix GeneChip® Human Mapping 500K Array Set | Broad Institute | BRLMM |
| **DIL** | rs3751812 | T | G | 0.40 | 0.77 | 0.999 | 0 | De novo /In Silico (IC;ICQ; OQ; Metabo) | TaqMan & rs9939609 | Wellcome Trust Sanger Institute | Applied Biosystems SDS |
| **EGCUT** | rs9939609 | A | T | 0.44 | 0.56 | 1 | 0 | In silico | Illumina 318K, 370K, Affymetrix 250K | Erasmus MC | BEADSTUDIO, BRLMM |
| **ERF** | rs9939609 | A | T | 0.43 | NA | NA | 1 | In silico | Illumina Human670-QuadCustom | Wellcome Trust Sanger Institute |  |
| **FINNTWIN12** | rs3751812 | T | G | 0.41 | 0.95 | 0.998 | 0 | In silico | Illumina Human670-QuadCustom | Wellcome Trust Sanger Institute |  |
| **FR02** | rs9939609 | A | T | 0.41 | 0.65 | 0.999 | 0 | De novo | Sequenom | Institute for Molecular Medicine Finland (FIMM) | Manually curated |
| **FR07** | rs9939609 | A | T | 0.39 | 0.19 | 0.999 | 0 | De novo | Sequenom | Institute for Molecular Medicine Finland (FIMM) | Manually curated |
| **FR92** | rs9939609 | A | T | 0.40 | 0.11 | 0.999 | 0 | De novo | Sequenom | Institute for Molecular Medicine Finland (FIMM) | Manually curated |
| **FR97** | rs9939609 | A | T | 0.40 | 0.63 | 0.999 | 0 | De novo | Sequenom | Institute for Molecular Medicine Finland (FIMM) | Manually curated |
| **FTC** | rs3751812 | T | G | 0.41 | 0.85 | 0.987 | 0 | In silico | Illumina Human670-QuadCustom | Wellcome Trust Sanger Institute |  |
| **GODARTSDIAB** | rs9939609 | A | T | 0.42 | 0.30 | 1 | 0 | In silico | Affymetrix 6.0/Cardio-Metabo BeadChip (Illumina 200K) | Sanger | CHIAMO/genosnp |
| **GODARTSNONDIAB** | rs9939609 | A | T | 0.40 | 0.13 | 1 | 0 | In silico | Affymetrix 6.0/Cardio-Metabo BeadChip (Illumina 200K) | Sanger | CHIAMO/genosnp |
| **GOSH** | rs9939609 | A | T | 0.39 | 0.24 | 1 | 0 | In silico | Cardio-Metabo BeadChip (Illumina 200K) | DeCode | GenCall algorithm, GenomeStudio, Illumina |
| **GRAPHIC** | rs9939609 | A | T | 0.40 | 0.04 | 1 | 0 | In silico | HumanCVD 50K BeadChip | Leicester | GenCall |
| **H2000** | rs9939609 | A | T | 0.40 | 0.51 | 0.993 | 0 | De novo | Sequenom | Institute for Molecular Medicine Finland (FIMM) | Manually curated |
| **KORAF3** | rs11075989 | T | C | 0.40 | 0.97 | 1 | 0 | In silico | Cardio-Metabo BeadChip (Illumina 200K) | Munich | GenCall algorithm, GenomeStudio, Illumina |
| **KORAF4** | rs11075989 | T | C | 0.40 | 0.97 | 0.999 | 0 | In silico | Cardio-Metabo BeadChip (Illumina 200K) | Munich | GenCall algorithm, GenomeStudio, Illumina |
| **MDCCV** | rs9939609 | A | T | 0.41 | 0.83 | 0.976 | 0 | De novo | Taqman Assay (Applied Biosystems) | Lund University Diabetes center |  |
| **MORGAM** | rs11075989 | T | C | 0.41 | 0.11 | 1 | 0 | In silico | Cardio-Metabo BeadChip (Illumina 200K) | Wellcome Trust Sanger Institute | GenCall algorithm, GenomeStudio, Illumina |
| **MPP** | rs9939609 | A | T | 0.41 | 0.92 | 0.99 | 0 | De novo | Taqman Assay (Applied Biosystems) | Lund University Diabetes Centre | SDS v 2.2 |
| **NESDA** | rs9939609 | A | T | 0.40 | NA | NA | 1 | In silico | Illumina 660K / Affymetrix (660K/907K) | Perlegen (USA), Tgene (USA) | Affymetrix proprietary, Birdsuite |
| **NFBC1966** | rs9939609 | A | T | 0.39 | 0.62 | 0.96 | 0 | De novo | TaqMan® SNP genotyping assay (Applied Biosystems, Warrington, UK) | Oxford Centre for Diabetes Endocrinology and Metabolism |  |
| **NFBC1986** | rs1421085 | C | T | 0.41 | 0.61 | 0.994 | 0 | De novo | Taqman Assay (Applied Biosystems) | Institute of Biology, Pasteur Institute, Lille, France, CNRS-UMR8090 | 7900 HT SDS 3.2 (Applied Biosystems) |
| **NTR** | rs9939609 | A | T | 0.38 | NA | NA | 1 | In silico | In silico: Illumina (907K/660K/370K) / Affymetrix (660K/1M); De novo: Sequenom MassARRAY iPLEX Platform | In silico: various; De novo: Moleculaire Epidemiologie, Leiden University | In silico: Affymetrix proprietary, Birdsuite; De novo: MassARRAY Analyzer 4 System |
| **PIVUS** | rs9939609 | A | T | 0.41 | 1 | 1 | 0 | In silico | Cardio-Metabo BeadChip (Illumina 200K) | Uppsala SNP Technology Platform | GenCall algorithm, GenomeStudio, Illumina |
| **PPP** | rs9939609 | A | T | 0.38 | 0.36 | 0.95 | 0 | De novo | Taqman Assay (Applied Biosystems) | Lund University Diabetes Centre | SDS v 2.2 |
| **QIMR-AUSTRALIA** | rs9939609 | A | T | 0.40 | NA | NA | 1 | In silico | Illumina 317K, Illumina 370K, Illumina 610K chip | University of Helsinki, DeCode |  |
| **RS** | rs9939609 | A | T | 0.38 | NA | NA | 1 | In silico | Version 3 Illumina Infinium II HumanHap 550 SNP chip array | Genetic Laboratory Dept Internal Medicine, Erasmus MC, The Netherlands | BeadStudio, Genecall |
| **TWINGENE** | rs9939609 | A | T | 0.41 | 0.52 | 1 | 0 | De novo | Centaurus (Nanogen) | DeCode |  |
| **TwinsUK** | rs3751812 | T | G | 0.39 | 0.70 | 1 | 0 | In silico | Illumina | WTSI,CIDR | Illluminus |
| **ULSAM** | rs9939609 | A | T | 0.39 | 0.24 | 1 | 0 | In silico | Cardio-Metabo BeadChip (Illumina 200K) | Uppsala SNP Technology Platform | GenCall algorithm, GenomeStudio, Illumina |
| **WTCCCCases** | rs11075989 | T | C | 0.41 | 0.52 | 1 | 0 | In silico | Cardio-Metabo BeadChip (Illumina 200K) | Sanger | GenCall algorithm, GenomeStudio, Illumina |
| **WTCCCCont** | rs11075989 | T | C | 0.39 | 0.76 | 1 | 0 | In silico | Cardio-Metabo BeadChip (Illumina 200K) | Sanger | GenCall algorithm, GenomeStudio, Illumina |
| **WTCCCT2D** | rs9939609 | A | T | 0.45 | 0.10 | 1 | 0 | In silico | Affymetrix / 500k (T2C cases) /6.0 (NBS & 58BC controls) | Oxford |  |
