# Supplementary material for: The Role of Adiposity in Cardiometabolic Traits: A Mendelian Randomization Analysis
Source: PLoS Med. 2013 Jun 25;10(6):e1001474. doi: 10.1371/journal.pmed.1001474 (PMC3692470; doi:10.1371/journal.pmed.1001474)
Supplement: Table S3 — List of proxies. (DOCX) [file pmed.1001474.s005.docx]

**Table S3**. **List of proxies with r2>0**.**9 for *FTO* variant rs9939609 at chr 16, position 52378028, alleles T/A**.

| **Proxies** | **Alleles on (+) strand** | **Position hg18** | **Distance from index SNP** | **r^2^ to best SNP** |
| --- | --- | --- | --- | --- |
| rs11075990 | A/G | 52377394 | 634 | 1 |
| rs11075989 | C/T | 52377378 | 650 | 1 |
| rs3751812 | G/T | 52375961 | 2067 | 1 |
| rs9935401 | G/A | 52374339 | 3689 | 1 |
| rs8051591 | A/G | 52374253 | 3775 | 1 |
| rs8050136 | C/A | 52373776 | 4252 | 1 |
| rs8043757 | A/T | 52370951 | 7077 | 1 |
| rs17817449 | T/G | 52370868 | 7160 | 1 |
| rs7202116 | A/G | 52379116 | 1088 | 0.967 |
| rs9923233 | C/G | 52376699 | 1329 | 0.967 |
| rs7185735 | A/G | 52380152 | 2124 | 0.967 |
| rs17817964 | C/T | 52385567 | 7539 | 0.967 |
| rs7193144 | T/C | 52368187 | 9841 | 0.967 |
| rs9936385 | T/C | 52376670 | 1358 | 0.935 |
| rs1558902 | T/A | 52361075 | 16953 | 0.934 |
| rs1421085 | T/C | 52358455 | 19573 | 0.934 |
| rs12149832 | G/A | 52400409 | 22381 | 0.934 |
