# Supplementary material for: The Role of Adiposity in Cardiometabolic Traits: A Mendelian Randomization Analysis
Source: PLoS Med. 2013 Jun 25;10(6):e1001474. doi: 10.1371/journal.pmed.1001474 (PMC3692470; doi:10.1371/journal.pmed.1001474)
Supplement: Table S4 — Definitions of outcomes and trait transformations. (DOCX) [file pmed.1001474.s006.docx]

**Table S4A. Definitions of outcomes**

| **Trait** | **Definition** | **Exclusion for logistic models** | **Exclusion for Cox regression models** |
| --- | --- | --- | --- |
| **Coronary heart disease (acute myocardial infarction or unstable angina)** | Either defined from hospital discharge registry or cause of death registry (main diagnosis); or from validated events ICD-8 codes: 410, 411 , ICD-9 codes: 410, 411B, ICD-10 codes: I20.0, I21, I22, *Note: Self-reported events are considered not useful.* | None | Free of any major CVD (coronary heart disease, stroke *and* heart failure) at BMI measurement, as defined in middle column |
| **Ischemic stroke** | Either defined from hospital discharge registry or cause of death registry (main diagnosis); or from adjudicated events ICD-8 codes: 432-434, ICD-9 codes: 433-434, ICD-10 codes: I63, *Note: Self-reported events are considered not useful.* | None | Free of any major CVD (coronary heart disease, stroke *and* heart failure) at BMI measurement, as defined in middle column |
| **Hemorrhagic stroke** | Either defined from hospital discharge registry or cause of death registry (main diagnosis); or from adjudicated events ICD-8 codes: 430-431, ICD-9 codes 430-432, ICD-10 codes: I60-I62, *Note: Self-reported events are considered not useful* | None | Free of any major CVD (coronary heart disease, stroke and heart failure) at BMI measurement, as defined in middle column |
| **Any acute stroke or transient ischemic attack** | Either defined from hospital discharge registry or cause of death registry (main diagnosis); or from adjudicated events ICD-8 codes: 430-436, ICD-9 codes: 430-436, ICD-10 codes: I60-I64+G45, *Note: Self-reported events are considered not useful* | None | Free of any major CVD (coronary heart disease, stroke and heart failure) at BMI measurement, as defined in middle column |
| **Heart failure** | Either defined from hospital discharge registry or cause of death registry (main diagnosis); or from adjudicated events, ICD-8 codes: 427.00, 427.10, ICD-9 codes: 428, ICD-10 codes: I50, *Note: Self-reported events are considered not useful* | None | Free of any major CVD (coronary heart disease, stroke and heart failure) at BMI measurement, as defined in middle column |
| **Type 2 diabetes** | Fasting blood glucose ≥7 mmol/L or anti-diabetic treatment. Self-reported diabetes | Type I diabetics, pregnant at blood sampling | Free of T2D at BMI measurement, as defined in middle column type 1 diabetes, pregnant at blood sampling |
| **Hypertension** | Systolic blood pressure ≥140, diastolic blood pressure ≥90, or on anti-hypertensive treatment | None | Free of hypertension at BMI measurement, as defined in middle column |
| **Dyslipidemia** | Serum triglycerides ≥1.7 mmol/L, HDL-cholesterol < 1.0 mmol/L in men and <1.3 mmol/L in women, or treatment with nicotinic acid or fibrates | None | Free of dyslipidemia at BMI measurement, as defined in middle column |
| **Metabolic Syndrome** | At least 3 out of 5 criteria should be present: | None | Free of METS at BMI measurement, as defined in middle column |
|  | . waist circumference (men, ≥102 cm; women, ≥88 cm), |  |  |
|  | . elevated triglycerides (≥1.7 mmol/l), |  |  |
|  | . reduced HDL cholesterol (men, <1.03 mmol/l; women, <1.29 mmol/l), |  |  |
|  | . blood pressure (systolic ≥130 mmHg or diastolic ≥85mmHg), |  |  |
|  | . fasting glucose (≥5.6 mmol/l). |  |  |

**Table S4B. Definitions of quantative outcomes and transformations**

| **Trait** | **Unit** | **transformation** | **exclusion** |
| --- | --- | --- | --- |
| **Glucose** | mmol/l | if measured in blood, convert to plasma scale | Exclude all individuals with diabetes (T2D, T1D) from datasets ('diagnosed', on diabetes treatment (oral and insulin), and/or FPG >=7 mmol/L), non-fasting or pregnant |
| **Insulin** | pmol/l | Natural log-transformed | Same as above |
| **HbA1c** | % | use the NGSP definition | Same as above |
|  |  |  | PLUS |
|  |  |  | Exclude samples with major blood abnormalities (thalassemia, sickle cell anemia, etc). |
|  |  |  | Exclude samples who have had a blood transfusion in the previous 2-3 months |
| **2h post OGTT glucose** | mmol/l |  | Same as for FG |
| **C-peptide** | nmol/l | Natural log-transformed | Same as for FG |
| **High-density-lipoprotein cholesterol** | mmol/l |  | Non-fasting, Patients on lipid-lowering medication |
| **Low-density-lipoprotein cholesterol** | mmol/l |  | Non-fasting, Patients on lipid-lowering medication |
| **Triglycerides** | mmol/l | Natural log-transformed | Non-fasting, Patients on lipid-lowering medication |
| **Total cholesterol** | mmol/l |  | Non-fasting, Patients on lipid-lowering medication |
| **Systolic blood pressure** | mmHg |  | None |
| **Diastolic blood pressure** | mmHg |  | None |
| **Alanine aminotransferase** | U/l | Natural log-transformed | Known liver disease |
| **Gamma-glutamyl transferase** | U/l | Natural log-transformed | Known liver disease |
| **Interleukin-6** | pg/ml | Natural log-transformed | Known inflammatory disease or acute infection (at time of blood sampling) |
| **C-reactive protein** | mg/l | Natural log-transformed, measured using high-sensitivity assays | Known inflammatory disease or acute infection (at time of blood sampling) |
