# Supplementary material for: The Role of Adiposity in Cardiometabolic Traits: A Mendelian Randomization Analysis
Source: PLoS Med. 2013 Jun 25;10(6):e1001474. doi: 10.1371/journal.pmed.1001474 (PMC3692470; doi:10.1371/journal.pmed.1001474)
Supplement: Table S5 — Specifications of assays used for quantitative traits and study-specific definitions of binary traits. (DOCX) [file pmed.1001474.s007.docx]

**Table S5. Specifications of assays used for quantitative traits and study-specific definitions of binary traits. (Part 1)**

|  | |  |  |  |  |  |
| --- | --- | --- | --- | --- | --- | --- |
| **COHORT** | **Glucose** | **Insulin** | **HbA1c** | **C-peptide** | **HDL-C** | **LDL-C** |
| **DECODE** | Roche diagnostics and Technicon auto-analyser or the Hitachi 912 clinical chemistry auto-analyser | Insulin electrochemiluminescence immunoassay | NA | NA | Enzymatic techniques. Data derived from 2 labs (Landspitali University Hospital and RAM). | Data derived from two labs (Landspitali University Hospital and RAM). Most data derived through Friedewald's formula. Minority directly measured. |
| **DGIcases** | Plasma glucose was measured with a glucose oxidase method (Beckman Glucose Analyzer, Beckman Instruments, Fullerton, CA) | Radioimmunoassay (Pharmacia, Uppsala, Sweden), enzyme linked immunoassay (DAKO Diagnostics Ltd, Cambridgeshire, UK), and fluoroimmunometric assay (AutoDelfia, Perkin Elmer Finland, Turku, Finland) | Local laboratories | Human C-peptide RIA-kit (Linco Research, St. Charles, Missouri, USA) | Cobas Mira analyzer (Hoffmann La Roche, Basel, Switzerland) in the Finnish cohort, Hitachi 911 (Boehringer Mannheim, Mannheim, Germany) in the Southern Swedish cohort and Technicon DAX 48 (Bayer Sweden AB, Gothenburg, Sweden) in the Skara cohort | Friedewald formula |
| **DGIcontrols** | Plasma glucose was measured with a glucose oxidase method (Beckman Glucose Analyzer, Beckman Instruments, Fullerton, CA) | Radioimmunoassay (Pharmacia, Uppsala, Sweden), enzyme linked immunoassay (DAKO Diagnostics Ltd, Cambridgeshire, UK), and fluoroimmunometric assay (AutoDelfia, Perkin Elmer Finland, Turku, Finland) | Local laboratories | Human C-peptide RIA-kit (Linco Research, St. Charles, Missouri, USA) | Cobas Mira analyzer (Hoffmann La Roche, Basel, Switzerland) in the Finnish cohort, Hitachi 911 (Boehringer Mannheim, Mannheim, Germany) in the Southern Swedish cohort and Technicon DAX 48 (Bayer Sweden AB, Gothenburg, Sweden) in the Skara cohort | Friedewald formula |
| **DIL** | NA | NA | ion exchange HPLC | NA | autoanalyser | NA |
| **EGCUT** | Reference method at Tartu University Hospital | NA | Reference method at Tartu University Hospital | NA | Reference method at Tartu University Hospital | Reference method at Tartu University Hospital |
| **ERF** | assay info na / measured in serum | assay info na / measured in serum | NA | NA | assay info na / measured in serum | NA |
| **FINNTWIN12** | NA | NA | NA | NA | NMR | NMR |
| **FTC** | NA | NA | NA | NA | NA | NA |
| **FR92** | NA | NA | NA | NA | Dextran-MgCl2 precipitation (average 4-hour fasting time) | LDL cholesterol was calculated using Friedewald's formula: LDL=serum cholesterol-HDL-(0.45.serum triglycerides), Average 4-hour fasting time. |
| **FR97** | NA | NA | NA | NA | Dextran-MgCl2 precipitation (average 4-hour fasting time) | LDL cholesterol was calculated using Friedewald's formula: LDL=serum cholesterol-HDL-(0.45.serum triglycerides), Average 4-hour fasting time. |
| **FR02** | NA | NA | NA | NA | Direct, polyethylene glycol-modified enzyme (PEG) (average 4-hour fasting time) | LDL cholesterol was calculated using Friedewald's formula: LDL=serum cholesterol-HDL-(0.45.serum triglycerides), Average 4-hour fasting time. |
| **FR07** | Enzymatic, hexokinase (fasting time >= 8 hours) | CMIA, Chemiluminescent Microparticle Immuno Assay (measured from serum) | NA | NA | Accelerator selective detergent | Lipid Selective Detergent |
| **GODARTS** | Gluco-quant Glucose / HK, Roche Diagnostics | ELISA assay (Mercodia) | Reference method at NHS Tayside | NA | Reference method at NHS Tayside | Friedewald calculated |
| **GOSH** | Reference method at Karolinska Institutet | Reference method at Karolinska Institutet | Reference method at Karolinska Institutet | NA | Reference method at Karolinska Institutet | Reference method at Karolinska Institutet |
| **GRAPHIC** | NA | NA | NA | NA | Abbott Aeroset 2.0 Analyser | Abbott Aeroset 2.0 Analyser |
| **H2000** | Glucose, Hexokinase (4-11 hour fasting time) | Microparticle enzyme immunoassay (4-11 hour fasting time) | NA | NA | HDL-C Plus (4-11 hour fasting time) | LDL-C Plus (4-11 hour fasting time) |
| **KORA F3** | GLU Flex (Dade Behring); Hexokinase/G6P-DH | NA | turbidimetric immunologic inhibition assay (TINIA; HA1C Kit Dade Behring) | NA | AHDL Flex method (Dade-Behring) | direct method (ALDL, Dade-Behring). |
| **KORA F4** | GLU Flex (Dade Behring); Hexokinase/G6P-DH | ELISA | HPLC (Menarini HA-8160) | NA | AHDL Flex method (Dade-Behring) | direct method (ALDL, Dade-Behring). |
| **MDCCV** | whole blood glucose, hexokinase method | radioimmunoassay | standars procedures (Dept Clincal Chemistry, Malmö University Hospital) | NA | standars procedures (Dept Clincal Chemistry, Malmö University Hospital) | Friedewalds |
| **MORGAM** | NA | NA | NA | NA | Dextran sulphate-Mg++ method, phosphotungstate-Mg++ method, or updated phosphotungstate-Mg++ method after the Boehringer-Ms. | Calculated using Friedewald's formula. |
| **MPP** | Hexokinase method (routine methods at the Department of Clinical Chemistry, University Hospital) | NA | routine methods at the Department of Clinical Chemistry, University Hospital | NA | routine methods at the Department of Clinical Chemistry, University Hospital | Friedewald formula |
| **NESDA** | Hexokinase method (Gluco-quant) (Modular analytics, Roche diagnostics, Mannheim, Germany) | NA | NA | NA | Enzymatic colorimetric assay (HDL-C plus) (Modular analytics, Roche diagnostics, Mannheim, Germany) | LDL cholesterol was calculated using Friedewald's formula (only if triglycerides < 5.0mmol/L): LDL=cholesterol-HDL-(0.45.triglycerides). |
| **NFBC1966** | Blood glucose was analysed by a glucose dehydrogenase method (Granutest 250, Diagnostica Merck, Darmstadt, Germany) | Serum insulin was analysed by RIA (Pharmacia Diagnostics, Uppsala, Sweden) | NA | NA | NA | Serum LDL was calculated by the Friedewald formula if the serum TG level was less than 354 mg/dL; if the TG level was greater than equal 354 mg/dL, LDL was determined by precipitating LD-lipoproteins with heparin and measuring cholesterol in the liquid phase and subtracting it from TC |
| **NFBC1986** | Plasma glucose concentrations were analysed by Cobas Integra 700 automatic analyser (Roche Diagnostics, Basel, Switzerland) | Serum insulin was determined by radioimmunoassay (Pharmacia Diagnostics, Uppsala, Sweden) | NA | NA | High-density lipoprotein (HDL)-cholesterol concentrations were analysed by Cobas Integra 700 automatic analyser (Roche Diagnostics, Basel, Switzerland) | Low-density lipoprotein (LDL)-cholesterol concentrations were analysed by Cobas Integra 700 automatic analyser (Roche Diagnostics, Basel, Switzerland) |
| **NTR** | Vitros 250 Glucose assay (Johnson & Johnson, Rochester, USA; measured in heparin plasma) | Immulite 1000 Insulin Method (Diagnostic Product Corporation, Los Angeles, USA; measured in heparin plasma) | Nyocard HbA1c assay (Axis-Shield, Oslo, Norway; measured in EDTA whole blood) | NA | Vitros 250 direct HDL cholesterol assay (Johnson & Johnson, Rochester, USA; measured in heparin plasma) | LDL cholesterol was calculated using Friedewald's formula: LDL=plasma cholesterol-HDL-(0.20.plasma triglycerides). |
| **PIVUS** | Reference method at Uppsala University Hospital | Enzymatic-immunological assay at Uppsala University Hospital | NA | NA | Reference method at Uppsala University Hospital | Reference method at Uppsala University Hospital |
| **PPP** | Glucose dehydrogenase method (Hemocue, Ängelholm, Sweden) | Serum insulin by fluoroimmunometric assay (Delfia, Perkin Elmer, Turku, Finland) | Local laboratories | Human C-peptide RIA-kit (Linco Research, St. Charles, Missouri, USA) | Enzymatic method (Konelab 60i analyser; Thermo Electron Oy, Vantaa, Finland) | Friedewald formula |
| **QIMR-AUSTRALIA** | NA | NA | NA | NA | Direct Assay, Roche Cholesterol Oxidase | Friedewald Calculation |
| **RS** | Glucose levels were measured using the glucose hexokinase method (Instruchemie) | Serum insulin was determined by metric assay (Biosource Diagnostics, Camarillo, CA, USA). | NA | NA | HDL-c was determined enzymatically, using an automated procedure | LDL cholesterol was calculated using Friedewald's formula: LDL=serum cholesterol-HDL-(0.45.serum triglycerides). |
| **TWINGENE** | Reference method at Karolinska Institutet | NA | Reference method at Karolinska Institutet | NA | Reference method at Karolinska Institutet | Reference method at Karolinska Institutet |
| **TwinsUK** | Ektachem 700 multichannel analyzer using an enzymatic colorimetric slide assay (Johnson and Johnson Clinical Diagnostic Systems, Amersham, U.K.) | immunoassay (Abbott Laboratories, Maidenhead, U.K.) | NA | NA | precipitation with magnesium chloride/phosphotumgstate and thereafter as TC | LDL cholesterol was calculated using Friedewald's formula: LDL=serum cholesterol-HDL-(0.45.serum triglycerides). |
| **ULSAM** | Glucose dehydrogenase method (Gluc-DH, Merck, Darmstadt, Germany) | Immunoreactive insulin: Enzymatic-immunological assay (Enzymun, Boehringer Mannheim) | HPLC with gradient system (BIO-RAD Laboratories) | NA | precipitation with magnesium chloride/phosphotumgstate and thereafter as TC | LDL cholesterol was calculated using Friedewald's formula: LDL=serum cholesterol-HDL-(0.45.serum triglycerides). |
| **WTCCCCont** | NA | NA | ion exchange HPLC | NA | Abbott Aeroset 2.0 Analyser | Abbott Aeroset 2.0 Analyser |
| **WTCCC Cases** | NA | NA | NA | NA | Abbott Aeroset 2.0 Analyser | Abbott Aeroset 2.0 Analyser |
| **WTCCCT2D** | NA | NA | NA | NA | NA | NA |

**Table S5. Specifications of assays used for quantitative traits and study-specific definitions of binary traits. (Part 2)**

| **COHORT** | **Triglycerides** | **Total cholesterol** | **Alanine aminotransferase (ALT)** | **Gamma-glytamyl-transferase (GGT)** | **Interleukine-6** | **CRP** |
| --- | --- | --- | --- | --- | --- | --- |
| **DECODE** | Enzymatic techniques. Data derived from 2 labs (Landspitali University Hospital and RAM). | Enzymatic techniques. Data derived from 2 labs (Landspitali University Hospital and RAM). | NA | NA | NA | Reference method at Landspitali University and RAM. |
| **DGIcases** | Cobas Mira analyzer (Hoffmann La Roche, Basel, Switzerland) in the Finnish cohort, Hitachi 911 (Boehringer Mannheim, Mannheim, Germany) in the Southern Swedish cohort and Technicon DAX 48 (Bayer Sweden AB, Gothenburg, Sweden) in the Skara cohort | Cobas Mira analyzer (Hoffmann La Roche, Basel, Switzerland) in the Finnish cohort, Hitachi 911 (Boehringer Mannheim, Mannheim, Germany) in the Southern Swedish cohort and Technicon DAX 48 (Bayer Sweden AB, Gothenburg, Sweden) in the Skara cohort | NA | NA | NA | NA |
| **DGIcontrols** | Cobas Mira analyzer (Hoffmann La Roche, Basel, Switzerland) in the Finnish cohort, Hitachi 911 (Boehringer Mannheim, Mannheim, Germany) in the Southern Swedish cohort and Technicon DAX 48 (Bayer Sweden AB, Gothenburg, Sweden) in the Skara cohort | Cobas Mira analyzer (Hoffmann La Roche, Basel, Switzerland) in the Finnish cohort, Hitachi 911 (Boehringer Mannheim, Mannheim, Germany) in the Southern Swedish cohort and Technicon DAX 48 (Bayer Sweden AB, Gothenburg, Sweden) in the Skara cohort | NA | NA | NA | NA |
| **DIL** | autoanalyser | autoanalyser | NA | NA | NA | nephelometry (Dade Behring) on citrated plasma samples after one thaw cycle. |
| **EGCUT** | Reference method at Tartu University Hospital | Reference method at Tartu University Hospital | Reference method at Tartu University Hospital | Reference method at Tartu University Hospital | NA | NA |
| **ERF** | assay info na / measured in serum | assay info na / measured in serum | NA | NA | NA | assay info na / measured in serum |
| **FINNTWIN12** | NMR | NMR | NA | NA | NA | NA |
| **FTC** | NA | NA | NA | NA | NA | NA |
| **FR92** | Enzymatic, GPO-PAP (average 4-hour fasting time) | Enzymatic, CHOD-PAP, (average 4-hour fasting time) | NA | NA | NA | NA |
| **FR97** | Enzymatic, GPO-PAP (average 4-hour fasting time) | Enzymatic, CHOD-PAP (average 4-hour fasting time) | NA | NA | NA | NA |
| **FR02** | Enzymatic, GPO-PAP (average 4-hour fasting time) | Enzymatic, CHOD-PAP (average 4-hour fasting time) | NA | NA | NA | NA |
| **FR07** | Enzymatic, GPO | Enzymatic, CHOD-PAP | NA | NA | NA | NA |
| **GODARTS** | Reference method at NHS Tayside | Reference method at NHS Tayside | NA | NA | NA | NA |
| **GOSH** | Reference method at Karolinska Institutet | Reference method at Karolinska Institutet | NA | Reference method at Karolinska Institutet | NA | Reference method at Karolinska Institutet |
| **GRAPHIC** | Abbott Aeroset 2.0 Analyser | Abbott Aeroset 2.0 Analyser | NA | NA | NA | NA |
| **H2000** | Triglycerides, GPO PAP (4-11 hour fasting time) | Cholesterol, CHOD PAP (4-11 hour fasting time) | NA | NA | NA | NA |
| **KORA F3** | TGL Flex (Dade-Behring), | cholesterol-esterase method (CHOL Flex, Dade-Behring) | UV test; IFCC with pyridoxal phosphate activation (Roche/Hitachi cobas) | Enzymatic calorimetric assay; HiCo Gamma-glutamyltransferase liquid (Roche/Hitachi cobas) against IFCC | sandwich ELISA (CLB, Amsterdam, The Netherlands) | high-sensitivity immunoradiometric assay (IMRA) |
| **KORA F4** | TGL Flex (Dade-Behring), | cholesterol-esterase method (CHOL Flex, Dade-Behring) | UV test; IFCC with pyridoxal phosphate activation (Roche/Hitachi cobas) | Enzymatic calorimetric assay; HiCo Gamma-glutamyltransferase liquid (Roche/Hitachi cobas) against IFCC | sandwich ELISA (CLB, Amsterdam, The Netherlands) | high-sensitivity immunoradiometric assay (IMRA) |
| **MDCCV** | standars procedures (Dept Clincal Chemistry, Malmö University Hospital) | standars procedures (Dept Clincal Chemistry, Malmö University Hospital) | NA | NA | NA | NA |
| **MORGAM** | NA | Dextran sulphate-Mg++ method, phosphotungstate-Mg++ method, or updated phosphotungstate-Mg++ method after the Boehringer-Ms. | NA | NA | NA | NA |
| **MPP** | routine methods at the Department of Clinical Chemistry, University Hospital | routine methods at the Department of Clinical Chemistry, University Hospital | NA | NA | NA | NA |
| **NESDA** | Enzymatic colorimetric assay (GPO-PAP) (Modular analytics, Roche diagnostics, Mannheim, Germany) | Enzymatic colorimetric assay (CHOD-PAP) (Modular analytics, Roche diagnostics, Mannheim, Germany) | NA | Enzymatic IFCC (Modular analytics, Roche diagnostics, Mannheim, Germany; measured in heparin plasma) | IL-6 ELISA HS (Pelikine Compact ELISA, Sanquin, Amsterdam, The Netherlands; measured in plasma) | CRP ELISA HS (Dako, Glostrup, Denmark; measured in plasma) |
| **NFBC1966** | Fasting serum triglycerides were determined using an Hitachi 911 automatic analyzer and commercial reagents (Roche, Mannheim, Germany) | Fasting serum total cholesterol was determined using an Hitachi 911 automatic analyzer and commercial reagents (Roche, Mannheim, Germany) | NA | NA | NA | Serum CRP concentrations were determined by immunoenzymometric assay (Medix Biochemica, Espoo, Finland) |
| **NFBC1986** | Triglyceride concentrations were analysed by Cobas Integra 700 automatic analyser (Roche Diagnostics, Basel, Switzerland) | Serum total cholesterol concentrations were analysed by Cobas Integra 700 automatic analyser (Roche Diagnostics, Basel, Switzerland) | NA | NA | NA | Serum CRP concentrations were determined by immunoenzymometric assay (Medix Biochemica, Espoo, Finland) |
| **NTR** | Vitros 250 Triglycerides assay (Johnson & Johnson, Rochester, USA; measured in heparin plasma) | Vitros 250 total cholesterol assay (Johnson & Johnson, Rochester, USA; measured in heparin plasma) | Vitros ALT assay (Johnson & Johnson, Rochester, USA; measured in heparin plasma) | Vitros GGT assay (Johnson & Johnson, Rochester, USA; measured in heparin plasma) | Quantikine human Interleukine-6 kit (R&D systems; measured in EDTA plasma) | Immulite 1000 CRP assay (Diagnostic Product Corporation, USA; measured in heparin plasma) |
| **PIVUS** | Reference method at Uppsala University Hospital | Reference method at Uppsala University Hospital | Reference method at Uppsala University Hospital | Reference method at Uppsala University Hospital | Evidence® array biochip analyser (Randox Laboratories Ltd, Crumlin, UK) | Ultra sensitive particle enhanced immunoturbidimetric assay (Orion Diagnostica, Espoo, Finland) |
| **PPP** | Enzymatic method (Konelab 60i analyser; Thermo Electron Oy, Vantaa, Finland) | Enzymatic method (Konelab 60i analyser; Thermo Electron Oy, Vantaa, Finland) | Local laboratories | NA | NA | NA |
| **QIMR-AUSTRALIA** | Enzymatic, Roche Method | Enzymatic, Roche Method | Roche, IFCC Method | Roche, IFCC Method | NA | NA |
| **RS** | Triglycerides were determined enzymatically, using an automated procedure | Total cholesterol was determined enzymatically, using an automated procedure | Automated biochemistry spectrophotometric analyzer (ELAN-Fully Selective Analyzer, Eppendorf- Merck, Hamburg, Germany) | Automated biochemistry spectrophotometric analyzer (ELAN-Fully Selective Analyzer, Eppendorf- Merck, Hamburg, Germany) | enzyme immuno assays according to the instructions of the manufacture (Medgenix, Amersfoort, the Netherlands). The lower detection limit of the assay was 3 pg/ml. | Rate Near Infrared Particle Immunoassay (Immage® Immunochemistry System, Beckman Coulter, USA). |
| **TWINGENE** | Reference method at Karolinska Institutet | Reference method at Karolinska Institutet | NA | NA | NA | Reference method at Karolinska Institutet, half of the cohort analyzed with "high-sensitive" assay. Models adjusted for 2 different methods |
| **TwinsUK** | colorimetric enzymatic method | colorimetric enzymatic method | kinetic rate method on a Synchron LX20 automated multi channel analyzer (Beckman Coulter, Fulleton, CA) multi channel analyzer (Beckman Coulter, Fulleton, CA) | kinetic rate method on a Synchron LX20 automated multi channel analyzer (Beckman Coulter, Fulleton, CA) multi channel analyzer (Beckman Coulter, Fulleton, CA) | hIL-6 Ultra-Sensitivity ELISA (BioSource, Nivelles, Belgium) | Human Cardiovascular Disease (CVD) Panel 2 (acute-phase proteins) LINCOplex Kit (HCVD2-67BK) from Linco (Millipore) and with the Extracellular Protein Buffer Reagent Kit (LHB0001) from Invitrogen |
| **ULSAM** | Enzymatic techniques using IL Test Cholesterol Trinders's Method and IL Test Enzymatic-colorimetric Method | Enzymatic techniques using IL Test Cholesterol Trinders's Method and IL Test Enzymatic-colorimetric Method | Greiner 300 analyser, enzymatic method | NA | IL-6 ELISA HS, R&D Systems, Minneapolis, MN | Latex enhanced reagent;Behring BN ProSpec analyzer |
| **WTCCCCont** | Abbott Aeroset 2.0 Analyser | Abbott Aeroset 2.0 Analyser | NA | NA | NA | nephelometry (Dade Behring) on citrated plasma samples after one thaw cycle. |
| **WTCCC Cases** | Abbott Aeroset 2.0 Analyser | Abbott Aeroset 2.0 Analyser | NA | NA | NA | NA |
| **WTCCCT2D** | NA | NA | NA | NA | NA | NA |

**Table S5. Specifications of assays used for quantitative traits and study-specific definitions of binary traits. (Part 3)**

| **COHORT** | **Coronary heart disease (acute myocardial infarction or unstable angina)** | **Ischemic stroke** | **Hemorrhagic stroke** | **Any acute stroke or transient ischemic attack** |
| --- | --- | --- | --- | --- |
| **DECODE** | registry-based (ICD9+10) | validated medical records | validated medical records | validated medical records |
| **DGIcases** | Registry-based | NA | NA | Registry-based |
| **DGIcontrols** | Registry-based | NA | NA | Registry-based |
| **DIL** | NA | NA | NA | NA |
| **EGCUT** | registry-based/self-reported (ICD10) | registry-based/self-reported (ICD10) | registry-based/self-reported (ICD10) | registry-based/self-reported (ICD10) |
| **ERF** | NA | NA | NA | NA |
| **FINNTWIN12** | NA | NA | NA | NA |
| **FTC** | NA | NA | NA | NA |
| **FR92** | Registry for social welfare and healthcare: I200, I21, I22 [ICD-10] / 410, 4110 [ICD-8/9]; causes of death registry: I20–I25, I46, R96, R98 [ICD-10] / 410-414, 798 (not 7980A) [ICD-8/9] | Registry for social welfare and healthcare: I63–I64 (not I636) [ICD -10] / 4330A, 4331A, 4339A, 4340A, 4341A, 4349A, 436 [ICD-9] / 433, 434, 436 [ICD-8] as S_PAADG, S_PAADGE, S_DG2, S_DG2E, S_DG3, S_DG3E, S_DG4 | NA | I60–I64 (not I636) [ICD -10] / 430, 431, 4330A, 4331A, 4339A, 4340A, 4341A, 4349A, 436 [ICD-9] / 430, 431 (except 43101, 43191) 433, 434, 436 [ICD-8] |
| **FR97** | Registry for social welfare and healthcare: I200, I21, I22 [ICD-10] / 410, 4110 [ICD-8/9]; causes of death registry: I20–I25, I46, R96, R98 [ICD-10] / 410-414, 798 (not 7980A) [ICD-8/9] | Registry for social welfare and healthcare: I63–I64 (not I636) [ICD -10] / 4330A, 4331A, 4339A, 4340A, 4341A, 4349A, 436 [ICD-9] / 433, 434, 436 [ICD-8] as S_PAADG, S_PAADGE, S_DG2, S_DG2E, S_DG3, S_DG3E, S_DG4 | NA | I60–I64 (not I636) [ICD -10] / 430, 431, 4330A, 4331A, 4339A, 4340A, 4341A, 4349A, 436 [ICD-9] / 430, 431 (except 43101, 43191) 433, 434, 436 [ICD-8] |
| **FR02** | Registry for social welfare and healthcare: I200, I21, I22 [ICD-10] / 410, 4110 [ICD-8/9]; causes of death registry: I20–I25, I46, R96, R98 [ICD-10] / 410-414, 798 (not 7980A) [ICD-8/9] | Registry for social welfare and healthcare: I63–I64 (not I636) [ICD -10] / 4330A, 4331A, 4339A, 4340A, 4341A, 4349A, 436 [ICD-9] / 433, 434, 436 [ICD-8] as S_PAADG, S_PAADGE, S_DG2, S_DG2E, S_DG3, S_DG3E, S_DG4 | NA | I60–I64 (not I636) [ICD -10] / 430, 431, 4330A, 4331A, 4339A, 4340A, 4341A, 4349A, 436 [ICD-9] / 430, 431 (except 43101, 43191) 433, 434, 436 [ICD-8] |
| **FR07** | Registry for social welfare and healthcare: I200, I21, I22 [ICD-10] / 410, 4110 [ICD-8/9]; causes of death registry: I20–I25, I46, R96, R98 [ICD-10] / 410-414, 798 (not 7980A) [ICD-8/9] | Registry for social welfare and healthcare: I63–I64 (not I636) [ICD -10] / 4330A, 4331A, 4339A, 4340A, 4341A, 4349A, 436 [ICD-9] / 433, 434, 436 [ICD-8] as S_PAADG, S_PAADGE, S_DG2, S_DG2E, S_DG3, S_DG3E, S_DG4 | NA | I60–I64 (not I636) [ICD -10] / 430, 431, 4330A, 4331A, 4339A, 4340A, 4341A, 4349A, 436 [ICD-9] / 430, 431 (except 43101, 43191) 433, 434, 436 [ICD-8] |
| **GODARTS** | registry-based (ICD9+10) | registry-based (ICD9+10) | registry-based (ICD9+10) | registry-based (ICD9+10) |
| **GOSH** | registry-based (ICD9+10) | registry-based (ICD9+10) | registry-based (ICD9+10) | registry-based (ICD9+10) |
| **GRAPHIC** | NA | NA | NA | NA |
| **H2000** | Registry for social welfare and healthcare: I200, I21, I22 [ICD-10] / 410, 4110 [ICD-8/9]; causes of death registry: I20–I25, I46, R96, R98 [ICD-10] / 410-414, 798 (not 7980A) [ICD-8/9] | Registry for social welfare and healthcare: I63–I64 (not I636) [ICD -10] / 4330A, 4331A, 4339A, 4340A, 4341A, 4349A, 436 [ICD-9] / 433, 434, 436 [ICD-8] as S_PAADG, S_PAADGE, S_DG2, S_DG2E, S_DG3, S_DG3E, S_DG4 | NA | I60–I64 (not I636) [ICD -10] / 430, 431, 4330A, 4331A, 4339A, 4340A, 4341A, 4349A, 436 [ICD-9] / 430, 431 (except 43101, 43191) 433, 434, 436 [ICD-8] |
| **KORA F3** | NA | NA | NA | NA |
| **KORA F4** | NA | NA | NA | NA |
| **MDCCV** | NA | NA | NA | NA |
| **MORGAM** | registry-based (ICD9+10) | NA | NA | registry-based (ICD9+10) |
| **MPP** | Registry-based | NA | NA | Registry-based |
| **NESDA** | NA | NA | NA | NA |
| **NFBC1966** | registry-based (ICD8+9+10) | registry-based (ICD8+9+10) | registry-based (ICD8+9+10) | registry-based (ICD8+9+10) |
| **NFBC1986** | registry-based (ICD8+9+10) | registry-based (ICD8+9+10) | registry-based (ICD8+9+10) | registry-based (ICD8+9+10) |
| **NTR** | NA | NA | NA | NA |
| **PIVUS** | validated medical records | NA | NA | validated medical records |
| **PPP** | Registry-based | NA | NA | Registry-based |
| **QIMR-AUSTRALIA** | NA | NA | NA | NA |
| **RS** | registry-based (ICD10) | NA | NA | adjudicated events based on medical records and neuroimaging |
| **TWINGENE** | registry-based (ICD9+10) | registry-based (ICD9+10) | registry-based (ICD9+10) | registry-based (ICD9+10) |
| **TwinsUK** | NA | NA | NA | NA |
| **ULSAM** | registry-based (ICD9+10) | registry-based (ICD9+10) | registry-based (ICD9+10) | registry-based (ICD9+10) |
| **WTCCCCont** | NA | NA | NA | NA |
| **WTCCC Cases** | NA | NA | NA | NA |
| **WTCCCT2D** | NA | NA | NA | NA |

**Table S5. Specifications of assays used for quantitative traits and study-specific definitions of binary traits. (Part 4)**

| **COHORT** | **Heart failure** | **Type 2 diabetes** | **Dyslipidemia** | **Hypertension** | **Metabolic Syndrome** |
| --- | --- | --- | --- | --- | --- |
| **DECODE** | NA | self-reported, diabetes medication and glucose measurements | NA | measurements and blood pressure medication | NA |
| **DGIcases** | NA | WHO 98 | lipid medication and lipid measurements | measurements and blood pressure medication | according to definition in document |
| **DGIcontrols** | NA | WHO 98 | lipid medication and lipid measurements | measurements and blood pressure medication | according to definition in document |
| **DIL** | NA | NA | NA | NA | NA |
| **EGCUT** | registry-based/self-reported (ICD10) | registry-based/self-reported (ICD10), diabetes medication and glucose measurements | registry-based/self-reported (ICD10), lipid medication and lipid measurements | registry-based/self-reported (ICD10), measurements and blood pressure medication | measurements |
| **ERF** | NA | glucose measurement and diabetes medication | lipid measurements and lipid medication | BP measurements and htn medication | measurements |
| **FINNTWIN12** | NA | NA | NA | NA | NA |
| **FTC** | NA | self-report of diabetes and type of treatment, pure insulin users excluded | NA | NA | NA |
| **FR92** | I50, I110, I130, I132 [ICD -10] / 4029B, 4148, 428 [ICD-9], 42700, 42710, 428 [ICD-8] | Self-reported or diagnosed T2D, fasting blood-glucose >= 7 or anti-diabetic treatment | Any type of lipid medication and lipid measurements | Measurements and blood pressure medication | NA |
| **FR97** | I50, I110, I130, I132 [ICD -10] / 4029B, 4148, 428 [ICD-9], 42700, 42710, 428 [ICD-8] | Self-reported or diagnosed T2D, fasting blood-glucose >= 7 or anti-diabetic treatment | Any type of lipid medication and lipid measurements | Measurements and blood pressure medication | NA |
| **FR02** | I50, I110, I130, I132 [ICD -10] / 4029B, 4148, 428 [ICD-9], 42700, 42710, 428 [ICD-8] | Self-reported or diagnosed T2D, fasting blood-glucose >= 7 or anti-diabetic treatment | Any type of lipid medication and lipid measurements | Measurements and blood pressure medication | NA |
| **FR07** | I50, I110, I130, I132 [ICD -10] / 4029B, 4148, 428 [ICD-9], 42700, 42710, 428 [ICD-8] | Self-reported or diagnosed T2D, fasting blood-glucose >= 7 or anti-diabetic treatment | Any type of lipid medication and lipid measurements | Measurements and blood pressure medication | ATP III |
| **GODARTS** | registry-based (ICD9+10) | validated journal study | NA | NA | NA |
| **GOSH** | registry-based (ICD9+10) | self-reported, diabetes medication and glucose measurements | lipid medication and lipid measurements | measurements and blood pressure medication | measurements |
| **GRAPHIC** | NA | Self reported | NA | BP measures or treatment | NA |
| **H2000** | I50, I110, I130, I132 [ICD -10] / 4029B, 4148, 428 [ICD-9], 42700, 42710, 428 [ICD-8] | Self-reported or diagnosed T2D, fasting blood-glucose >= 7 or anti-diabetic treatment | Any type of lipid medication and lipid measurements | Measurements and blood pressure medication | NA |
| **KORA F3** | NA | Diabetes definition based on self-reported diabetes or diabetic treatment | Serum triglycerides ≥1.7 mmol/L and HDL-cholesterol < 1.0 mmol/L in men and <1.3 mmol/L in women, or treatment with fibrates. For KORA F4 nearly all individuals were fasting and for KORA F3 most individuals were non fasting. We do not use fasting individuals only for definition of dyslepidemia | Systolic blood pressure ≥140, diastolic blood pressure ≥90, or on anti-hypertensive treatment | NA |
| **KORA F4** | NA | Diabetes definition based on self-reported diabetes or diabetic treatment | Serum triglycerides ≥1.7 mmol/L and HDL-cholesterol < 1.0 mmol/L in men and <1.3 mmol/L in women, or treatment with fibrates. For KORA F4 nearly all individuals were fasting and for KORA F3 most individuals were non fasting. We do not use fasting individuals only for definition of dyslepidemia | Systolic blood pressure ≥140, diastolic blood pressure ≥90, or on anti-hypertensive treatment | NCEP definition (lipids and glucose in mg/dl): At least 3 out of 5 criteria should be present: . waist circumference (men, =102 cm; women, =88 cm), . elevated triglycerides (=150 mg/dl), . reduced HDL cholesterol (men, <40 mg/dl; women, <50 mg/dl), . blood pressure (systolic =130 mmHg or diastolic =85mmHg), . fasting glucose (=110,g/dl). |
| **MDCCV** | NA | selfreported diagnosis, self-reported medication or measured fasting glucose <7 | selfreported lipid medication or lipid measurements | measurements or antihypertensive medication | measurements |
| **MORGAM** | NA | self-reported | lipid measurements | measurements of blood pressure | NA |
| **MPP** | NA | NA | NA | measurements and blood pressure medication | according to definition in document |
| **NESDA** | NA | self-reported, diabetes medication and glucose>=7 mmol/L (no distinction between type I and type II) | lipid medication and lipid measurements | NA | NA |
| **NFBC1966** | registry-based (ICD8+9+10) | self-reported, diabetes medication and glucose measurements | lipid measurements | measurements and blood pressure medication | measurements |
| **NFBC1986** | registry-based (ICD8+9+10) | diabetes medication and glucose measurements | lipid measurements | measurements | measurements |
| **NTR** | NA | medication or glucose >= 7 | medication or cholesterol profile | NA | NA |
| **PIVUS** | NA | self-reported, diabetes medication and glucose measurements | lipid medication and lipid measurements | measurements and blood pressure medication | measurements |
| **PPP** | NA | WHO 98 | lipid medication and lipid measurements | measurements and blood pressure medication | according to definition in document |
| **QIMR-AUSTRALIA** | NA | NA | NA | NA | NA |
| **RS** | registry-based (ICD10) | Diabetes medication and abnormal fasting or random glucose | lipid medication and lipid measurements | measurements and blood pressure medication | measurements |
| **TWINGENE** | registry-based (ICD9+10) | self-reported, diabetes medication and glucose measurements | lipid medication and lipid measurements | measurements and blood pressure medication | measurements |
| **TwinsUK** | NA | self-reported, diabetes medication and glucose measurements (OGTT) | lipid medication and lipid measurements | measurements and blood pressure medication | measurements |
| **ULSAM** | registry-based (ICD9+10) | self-reported, diabetes medication and glucose measurements (OGTT) | lipid medication and lipid measurements | measurements and blood pressure medication | measurements |
| **WTCCCCont** | NA | Self reported | NA | BP measures or treatment | NA |
| **WTCCC Cases** | NA | Self reported | NA | BP measures or treatment | NA |
| **WTCCCT2D** | NA | Validation of the diagnosis of diabetes in the index sib pair was based on either current prescribed treatment with sulfonyl ureas, biguanides, and/or insulin or, in the case of individuals treated with diet alone, historical or contemporary laboratory evidence of hyperglycemia (as deﬁned by World Health Organization [1985] guidelines in place at the time of recruitment). Diagnosis between age 25 and 75 | NA | NA | NA |
