# Supplementary material for: The Role of Adiposity in Cardiometabolic Traits: A Mendelian Randomization Analysis
Source: PLoS Med. 2013 Jun 25;10(6):e1001474. doi: 10.1371/journal.pmed.1001474 (PMC3692470; doi:10.1371/journal.pmed.1001474)
Supplement: Table S6 — Summary of sensitivity analysis for the effect of correlation on instrumental variable estimates. (DOCX) [file pmed.1001474.s008.docx]

**Table S6**. **Confidence intervals (CIs) for the estimates for different traits (on the logit/log scale for binary traits)**. **The delta method corresponds to the CI reported in Tables 1 and 2 of the paper**. **The plugin CI is based on the between-cohort estimate of the correlation between β[*FTO*-TRAIT] and β[*FTO*-BMI]**. **The worst case CI is based on the widest CI possible under any correlation**. **F1 is the length of the plugin CI divided by the length of the reported CI; F2 is the length of the worst case CI divided by the reported CI (all on the regression scale)**.

|  | **Delta method** | **Plug-in** | **Worst case** | **F1** | **F2** |
| --- | --- | --- | --- | --- | --- |
| **Ever coronary heart disease** | -0.129/0.119 | -0.136/0.114 | -0.148/0.105 | 1.01 | 1.02 |
| **Incident coronary heart disease** | -0.15/0.121 | -0.156/0.118 | -0.172/0.107 | 1.01 | 1.03 |
| **Ever heart failure** | 0.0432/0.276 | 0.0457/0.274 | 0.0399/0.315 | 0.98 | 1.18 |
| **Incident heart failure** | 0.0242/0.326 | 0.0261/0.328 | 0.023/0.372 | 1.00 | 1.16 |
| **Ever haemorrhagic stroke** | -0.421/0.334 | -0.429/0.333 | -0.483/0.295 | 1.01 | 1.03 |
| **Incident haemorrhagic stroke** | -1.03/0.219 | -1.02/0.223 | -1.18/0.192 | 0.99 | 1.10 |
| **Ever ischemic stroke** | -0.161/0.117 | -0.159/0.12 | -0.185/0.103 | 1.00 | 1.04 |
| **Incident ischemic stroke** | -0.131/0.312 | -0.125/0.333 | -0.116/0.358 | 1.03 | 1.07 |
| **Ever stroke** | -0.144/0.127 | -0.14/0.134 | -0.166/0.113 | 1.01 | 1.03 |
| **Incident stroke** | -0.142/0.229 | -0.134/0.247 | -0.125/0.263 | 1.03 | 1.05 |
| **Ever type 2 diabetes** | 0.21/0.414 | 0.228/0.396 | 0.193/0.466 | 0.82 | 1.34 |
| **Incident type 2 diabetes** | 0.116/0.48 | 0.137/0.424 | 0.106/0.546 | 0.79 | 1.21 |
| **Ever dyslipidaemia** | 0.0686/0.188 | 0.0719/0.186 | 0.0629/0.213 | 0.96 | 1.26 |
| **Incident dyslipidaemia** | -0.43/0.627 | - | -0.381/0.72 | - | 1.04 |
| **Ever hypertension** | 0.068/0.173 | 0.0706/0.173 | 0.0623/0.196 | 0.97 | 1.27 |
| **Incident hypertension** | -0.243/0.419 | - | -0.215/0.481 | - | 1.05 |
| **Ever metabolic syndrome** | 0.165/0.368 | 0.161/0.393 | 0.152/0.415 | 1.15 | 1.30 |
| **Incident metabolic syndrome** | -0.0173/0.723 | - | -0.0129/0.827 | - | 1.13 |
| **Incident mortality** | -0.103/0.0685 | -0.104/0.0687 | -0.118/0.0607 | 1.01 | 1.04 |
| **2h post OGTT glucose** | 0.0128/0.162 | 0.0129/0.172 | 0.0121/0.185 | 1.07 | 1.16 |
| **Fasting glucose** | -0.00446/0.0393 | -0.00429/0.0404 | -0.00385/0.045 | 1.02 | 1.12 |
| **HbA1c** | -0.0129/0.0269 | -0.0133/0.0264 | -0.0114/0.0308 | 1.00 | 1.06 |
| **Fasting insulin** | 0.0358/0.076 | 0.0379/0.0743 | 0.0329/0.0857 | 0.90 | 1.31 |
| **Diastolic blood pressure** | 0.187/0.793 | 0.197/0.786 | 0.172/0.903 | 0.97 | 1.21 |
| **Systolic blood pressure** | 0.475/1.31 | 0.497/1.3 | 0.435/1.48 | 0.96 | 1.26 |
| **HDL-C** | -0.0261/-0.00941 | -0.0257/-0.0099 | -0.0295/-0.00861 | 0.95 | 1.26 |
| **LDL-C** | -0.0122/0.0346 | -0.0124/0.0344 | -0.0107/0.0396 | 1.00 | 1.08 |
| **ALT** | 0.0161/0.0517 | 0.0162/0.0536 | 0.0147/0.0588 | 1.05 | 1.24 |
| **CRP** | 0.0343/0.1 | 0.0359/0.0994 | 0.0314/0.114 | 0.97 | 1.25 |
| **GGT** | 0.0187/0.0547 | 0.0214/0.0497 | 0.0172/0.0621 | 0.79 | 1.25 |
| **IL-6** | -0.0517/0.0615 | -0.0507/0.0639 | -0.0458/0.0707 | 1.01 | 1.03 |
| **Triglycerides** | 0.0162/0.0403 | 0.0172/0.0396 | 0.0149/0.0457 | 0.93 | 1.28 |
| **Total cholesterol** | -0.0177/0.0296 | -0.0176/0.0301 | -0.0157/0.0339 | 1.01 | 1.05 |
