# Supplementary material for: The Role of Adiposity in Cardiometabolic Traits: A Mendelian Randomization Analysis
Source: PLoS Med. 2013 Jun 25;10(6):e1001474. doi: 10.1371/journal.pmed.1001474 (PMC3692470; doi:10.1371/journal.pmed.1001474)
Supplement: Table S7 — Summary of sensitivity analysis for the effect of correlation on the estimates of differences between instrumental variable and observational analysis. (DOCX) [file pmed.1001474.s009.docx]

**Table S7**. **Confidence intervals (CIs) for the difference between the instrumental variable estimates β[IV] and the conventional regression estimates β[BMI_TRAIT] for different traits (on the logit/log scale for D/ITraits)**. **The delta method corresponds to the CI reported in Tables 1 and 2 of the paper**. **The plugin CI is based on the between-cohort estimate of correlations**. **The worst case CI is based on the widest CI possible under any correlation**. **F1 is the length of the plugin CI divided by the length of the reported CI; F2 is the length of the worst case CI divided by the reported CI (all on the regression scale)**.

|  | **Delta method** | **Plug-in** | **Worst case** | **F1** | **F2** |
| --- | --- | --- | --- | --- | --- |
| **Ever coronary heart disease** | -0.159/0.0907 | -0.164/0.0956 | -0.178/0.109 | 1.04 | 1.15 |
| **Incident coronary heart disease** | -0.196/0.0767 | -0.196/0.0768 | -0.213/0.094 | 1.00 | 1.13 |
| **Ever heart failure** | -0.041/0.197 | -0.0523/0.208 | -0.0801/0.236 | 1.10 | 1.33 |
| **Incident heart failure** | -0.0694/0.234 | -0.0755/0.24 | -0.107/0.271 | 1.04 | 1.25 |
| **Ever haemorrhagic stroke** | -0.409/0.348 | -0.407/0.345 | -0.447/0.386 | 0.99 | 1.10 |
| **Incident haemorrhagic stroke** | -1.02/0.233 | -0.997/0.206 | -1.13/0.34 | 0.96 | 1.17 |
| **Ever ischemic stroke** | -0.186/0.0944 | -0.2/0.108 | -0.209/0.117 | 1.10 | 1.16 |
| **Incident ischemic stroke** | -0.166/0.28 | -0.183/0.297 | -0.2/0.314 | 1.08 | 1.15 |
| **Ever stroke** | -0.157/0.117 | -0.164/0.123 | -0.177/0.137 | 1.05 | 1.15 |
| **Incident stroke** | -0.166/0.206 | -0.169/0.209 | -0.19/0.229 | 1.01 | 1.13 |
| **Ever type 2 diabetes** | 0.0682/0.274 | 0.0897/0.253 | 0.0237/0.319 | 0.79 | 1.43 |
| **Incident type 2 diabetes** | -0.0329/0.332 | 0.0202/0.279 | -0.0837/0.383 | 0.71 | 1.28 |
| **Ever dyslipidaemia** | -0.0739/0.0513 | -0.0659/0.0432 | -0.102/0.0798 | 0.87 | 1.46 |
| **Incident dyslipidaemia** | -0.481/0.56 | - | -0.543/0.621 | - | 1.12 |
| **Ever hypertension** | -0.0522/0.0553 | -0.051/0.0542 | -0.0742/0.0773 | 0.98 | 1.41 |
| **Incident hypertension** | -0.28/0.372 | - | -0.323/0.414 | - | 1.13 |
| **Ever metabolic syndrome** | -0.117/0.0935 | -0.132/0.108 | -0.168/0.144 | 1.14 | 1.48 |
| **Incident metabolic syndrome** | -0.206/0.52 | - | -0.278/0.592 | - | 1.20 |
| **Incident mortality** | -0.119/0.0546 | -0.127/0.0619 | -0.136/0.071 | 1.08 | 1.19 |
| **2h post OGTT glucose** | -0.0532/0.104 | -0.0671/0.118 | -0.0832/0.134 | 1.18 | 1.38 |
| **Fasting glucose** | -0.0331/0.0116 | -0.0359/0.0143 | -0.0394/0.0179 | 1.12 | 1.28 |
| **HbA1c** | -0.0357/0.00658 | -0.0382/0.00904 | -0.0426/0.0134 | 1.12 | 1.32 |
| **Fasting insulin** | -0.0249/0.0164 | -0.0234/0.015 | -0.0343/0.0259 | 0.93 | 1.46 |
| **Diastolic blood pressure** | -0.439/0.181 | -0.419/0.16 | -0.552/0.293 | 0.93 | 1.36 |
| **Systolic blood pressure** | -0.439/0.418 | -0.396/0.375 | -0.615/0.594 | 0.90 | 1.41 |
| **HDL-C** | -0.0038/0.0133 | -0.00298/0.0125 | -0.00732/0.0168 | 0.90 | 1.41 |
| **LDL-C** | -0.0305/0.0172 | -0.0327/0.0193 | -0.0363/0.023 | 1.09 | 1.24 |
| **ALT** | -0.0117/0.0264 | -0.0136/0.0283 | -0.0203/0.0351 | 1.10 | 1.46 |
| **CRP** | -0.0531/0.0228 | -0.0554/0.0251 | -0.0726/0.0423 | 1.06 | 1.51 |
| **GGT** | -0.0138/0.0231 | -0.0115/0.0208 | -0.0213/0.0306 | 0.87 | 1.40 |
| **IL-6** | -0.0863/0.0279 | -0.0827/0.0243 | -0.0944/0.0361 | 0.94 | 1.14 |
| **Triglycerides** | -0.0177/0.00685 | -0.0164/0.00557 | -0.0226/0.0118 | 0.90 | 1.40 |
| **Total cholesterol** | -0.0342/0.014 | -0.036/0.0158 | -0.0394/0.0193 | 1.07 | 1.22 |
