# Supplementary material for: The Role of Adiposity in Cardiometabolic Traits: A Mendelian Randomization Analysis
Source: PLoS Med. 2013 Jun 25;10(6):e1001474. doi: 10.1371/journal.pmed.1001474 (PMC3692470; doi:10.1371/journal.pmed.1001474)
Supplement: Text S1 — Extended methods. (DOCX) [file pmed.1001474.s010.docx]

### **Text S1.Extended Methods**

### **Software used for imputation in studies** Standard software packages IMPUTE and MACH were used for imputation[[1](#_ENREF_1),[2](#_ENREF_2)].

### **Per-study analysis: motivation**

For modelling the relationships between *FTO* genotype, the intermediate phenotype BMI and the different cardiometabolic traits as seen in Figure 1 of the manuscript, we distinguish between continuously scaled or quantitative traits (QTraits), dichotomous outcomes (DTraits, marked as “Ever” in Table 1 of the manuscript) and incident or survival outcomes (ITraits, marked as “Incident” in Table 1). Except for overall mortality, each ITrait has a corresponding DTrait in this study. We model the relationship between any predictor variable (*FTO* or BMI) and a QTrait as outcome via linear regression (possibly after logarithmizing the QTrait); for DTraits, we use logistic regression and for ITraits, Cox proportional hazard regression, as detailed below.

The purpose of the parallel analysis of prevalent and incident cases is to combine the benefits of the two approaches: survival analysis exploits time-to-event data more thoroughly and is easier to interpret causally, but is only available for a subset of cohorts for each of the D/ITraits; logistic regression ignores time scale, but is applicable to all cohorts with prevalent data, and has consequently more power to detect significant associations. For our study, the results from both approaches support each other well, with close effect estimates throughout (Table 1 of the manuscript).

Model definitions for per-cohort analysis
The following models were used to assess the relationship between (a) *FTO* and BMI, (b) BMI and traits, and (c) between *FTO* and traits. *Study specific covariates* below include, among others, study centres, sub-cohorts or principal components for population stratification.

(a) Analysis of association between *FTO* and BMI. Model: *linear regression*

BMI = SNP + sex + age + other study specific covariates
Age was defined as the age at baseline (time of the BMI measurement).

(b) Analysis of associations between BMI and the traits

(b.1) Quantitative traits (QTrait). Model: *linear regression*

QTrait = BMI+ sex + age+ other study specific covariates
Age was defined as the age at the time of measurement of the QTrait.

(b.2) Dichotomous traits (DTrait). Model: *logistic regression*

DTrait = BMI + sex + age + other study specific covariates

Age was defined as the age at event for DTrait in those who ever have the event, whereas in non-affected individuals, age was the latest known age (age at death or loss-to-follow-up).

(b.3) Incidence traits (ITrait). Model: *Cox proportional hazard regression*

ITrait = BMI+ sex [+ age at baseline] + other study specific covariates

Baseline refered to the time of BMI measurement. Individuals that did not reach the endpoint were censored at time of death or other loss-of-follow up.

(c) Analysis of *FTO* and outcome trait association with and without BMI adjustment

(c.1) Quantitative traits (QTrait). Model: *linear regression*

QTrait = SNP + sex + age + other study specific covariates
Age was defined as the age at the time of measurement of the QTrait.

(c.2) Dichotomous traits (DTrait). Model: *logistic regression*

DTrait = SNP + sex + age + other study specific covariates

Age was defined as the age at event for DTrait in those who ever have the event, whereas in non-affected individuals age was the latest known age (age at death or loss-to-follow-up).

(c.3) Incidence traits (ITrait). Model: *Cox proportional hazard regression*

ITrait = SNP + sex + age at baseline + other study specific covariates
Baseline refered to the time of BMI measurement.

Quality Control of Cohort-Specific Results

All genotypes were inspected for strand, lead- or proxy marker and effect allele reported. For all regression results, inverse standard errors were plotted by trait over the square-root of effective sample size [eff_n = 2/((1/n_cases)+(1/n_controls))] to ensure absence of trait transformation errors.

Meta-analysis and between-study heterogeneity

We assessed between-cohort heterogeneity via Cochrane’s Q-statistic and I^2^-statistics.[[3-5](#_ENREF_3)] Histograms of the distribution of I^2^ in associations between BMI and traits and associations between *FTO* and traits are shown in Panels A and C of Figure S1. For the proposed cut-off of I^2^>0.25, we found non-negligible heterogeneity between studies for many associations, in particular among the BMI-trait associations, but also for the association between *FTO* and BMI (I2 = 0.55). As a consequence, we used random-effects meta-analysis throughout. The results presented in Tables 1 and 2 of the manuscript are estimates of the *average* effect of BMI on the traits investigated rather than the common effect.[[6](#_ENREF_6)]

Different reasons for the variation between cohorts are possible: the relationship between BMI and cardiometabolic traits is of course famously confounded with other genetic and life style factors that vary systematically between cohorts. This may be driven by varying subject age, sex and disease distribution between contributing cohorts, and by differences in the outcome measurement methods among contributing cohorts. We analysed our aggregate data with meta-regression of betas with mean age as a covariate to assess the impact of cohort age on the effect sizes of *FTO*-trait and BMI-trait. The results show that [BMI-trait] betas are significantly (p<0.01) decreasing with cohort mean age for LDL cholesterol, triglycerides and total cholesterol. On the other hand, when examining the effect of cohort age on the *FTO*-trait betas, no effect was observed.

Figure S1 also shows the effect of choosing the random-effects model instead of the fixed-effects model on the Wald-statistics for both the IV estimator and the conventional regression estimator (Panel B): for the IV estimates, we see moderate shrinkage of the Wald test statistics, with only fasting glucose (labelled as FG) being significant at α=0.05 for fixed, but not random effects; arguably, even though the random effects model leads to slightly more conservative estimates, both models indicate comparable strength of evidence, in good agreement with the moderate I^2^ values seen in the corresponding histogram in Panel A, and the consistent estimate for the *FTO*-BMI association ($\beta_{FTO-BMI}=0.36$ under both fixed and random effects). This effect size is similar to earlier reported effect sizes of *FTO* on BMI.[[7](#_ENREF_7)]

For the estimates of BMI with trait, however, the use of the random effects model leads to severe shrinkage of the Wald test statistics and a reduction of their range by a factor of four in Panel D. This is clearly driven by the large amount of between-cohort variability shown in the corresponding histogram of I^2^ values in Panel C. Here, the use of the fixed effect estimates would overstate the strength of our case significantly, and this is the most pressing reason for our using the random effects model throughout.

Standard errors and inference for the instrumental variable (IV) estimator

After meta-analysis, the IV estimator for the effect of BMI on a trait was calculated as the ratio of the regression coefficients *FTO*-TRAIT and *FTO*-BMI:

|  | $\beta_{IV}=\frac{\beta_{FTO\_TRAIT}}{\beta_{FTO\_BMI}}$ |  |
| --- | --- | --- |

The standard error is calculated via the delta method as

|  | ${se}_{IV}\text{=}\left. abs\left( \beta_{IV} \right)\sqrt{\left( \frac{{se}_{FTO\_BMI}}{\beta_{FTO\_BMI}} \right)^{2}+\left( \frac{{se}_{FTO\_TRAIT}}{\beta_{FTO\_TRAIT}} \right)^{2}} \right.$ |  |
| --- | --- | --- |

Based on these estimates, we appeal to standard-normal asymptotics, with the resulting Wald test statistic and 95% confidence intervals given as

$t_{IV}=\frac{\beta_{IV}}{{se}_{IV}}$ ${CI}_{IV}=\beta_{IV}\pm1.96 {se}_{IV}$

The p-value for the $H_{0}$: $\beta_{IV}=0$ was derived from the standard normal distribution. For the odds ratios and hazard ratios associated with DTraits and ITraits, the 95% CI estimates were back-transformed through the antilogit and exponentiation, respectively.

For comparing the IV estimate $\beta_{IV}$ and the conventional estimate $\beta_{BMI\_TRAIT}$, we consider the difference

|  | $\beta_{Diff}=$ $\beta_{IV}- \beta_{BMI\_TRAIT}$ |  |
| --- | --- | --- |

The corresponding standard error is

|  | ${se}_{Diff}\text{=}\left. \sqrt{\left( {se}_{IV} \right)^{2}+\left( {se}_{BMI\_TRAIT} \right)^{2}} \right.$ |  |
| --- | --- | --- |

We use again standard normal asymptotics for the difference, viz.

$t_{Diff}=\frac{\beta_{Diff}}{{se}_{Diff}}$ ${CI}_{Diff}=\beta_{Diff}\pm1.96 {se}_{Diff}$

The p-value for the $H_{0}$: $\beta_{Diff}=0$ was derived from the standard normal distribution, and the confidence intervals for odds/hazard ratios are back-transformed as above.

#### Methodolocical considerations Apart from the assumptions described in the main paper, additional assumptions are required for unbiased causal estimates (providing quantification as opposed to testing only), specifically about the shape of the association between intermediate phenotype and outcome [[8](#_ENREF_8),[9](#_ENREF_9)]); we have assumed linearity throughout, for which there is reasonable evidence [[10](#_ENREF_10)]). Estimates are also sensitive towards epigenetic and GxE modifications of the relationship between *FTO* and BMI. There is preliminary evidence that environmental factors such as physical activity and fat intake may influence the association of *FTO* with BMI and potentially metabolic outcomes [4], but the observed effect amounts to only modest modification of the *FTO*-BMI relationship, and does not imply a direct association between *FTO* and physical activity/fat intake.

#### These possible interactions and epigenetic changes should not affect the causal interpretation per se, but could affect the precision of our estimates. Based on reported effect sizes of GxE-interactions, we expect the loss of power and the corresponding effect on causal estimates to be fairly small.

#### Sensitivity analysis for the IV inference

Correlations between the regression coefficients can affect the estimation of the standard errors for the quantities $\beta_{IV}$ and $\beta_{Diff}$ given in Eq. 1 and 3 above. The formulas for the standard errors underlying the results reported in the paper, given in Eq. 2 and 4, do not take this possibility into account. Given that the per-cohort regression coefficients underlying the meta-analysis are estimated on the same data, there is no *prima facie* reason to rule out that they are in fact correlated. As we do not have per-cohort estimates for the correlations between regression coefficients that would allow us to calculate a joint estimate through meta-analysis, we have evaluated the possible impact of such correlations through sensitivity analysis. Essentially, we compare the reported confidence intervals and p-values with two modified estimates:

1. Using the between-cohort estimate of the correlation between $\beta_{FTO\_TRAIT}$ and $\beta_{FTO\_BMI}$, and between $\beta_{IV}$ and $\beta_{BMI\_TRAIT}$, respectively, to adjust the confidence intervals. We refer to this as the meta-regression or *plugin*-estimate.
2. Using the widest confidence interval possible under any correlation in the interval ±1; we refer to this as the *worst-case* estimate.

For the IV estimator $\beta_{IV}$, we use Fieller's theorem to derive confidence limits that not only explicitly include the effect of correlation, but also provide a check on the quality of the delta method approximation.^5^Figure S2 shows a graphical summary of the results for the dichotomous trait “Ever heart failure” (variable HF_D), where the thick horizontal line represents the estimated effect $\beta_{IV}$ (on the logit scale); the broken horizontal lines show the 95% confidence interval based on the standard error given in Eq. 2, which is also reported in Table 1 of the paper (back-transformed via antilogit). These values are independent of the range of possible correlations between $\beta_{FTO\_TRAIT}$ and $\beta_{FTO\_BMI}$ shown on the x-axis of the plot, whereas the confidence interval based on Fieller’s theorem is shown as two curves that are more distant for negative correlations than for positive correlations. We find that:

1. the confidence interval for an assumed correlation of $r=0$ is almost identical to the interval arrived at via the delta method,
2. the plugin-estimate for the confidence interval, assuming that the observed correlation between $\beta_{FTO\_TRAIT}$ and $\beta_{FTO\_BMI}$ can be estimated via the between-cohort correlation of $r=0.14$ (shown as vertical thick grey line), is actually somewhat tighter than the reported confidence interval, and
3. the worst-case confidence interval can be seen at $r=-1$, and is ca. 30% wider than the reported CI; however, the extension is almost exclusively at the upper limit of the confidence interval, so the impact on comparing $\beta_{IV}$ with zero is negligible.

Corresponding plots for the other traits (not shown) look similar and lead to very similar conclusions. Table S6 shows a summary of the reported, the plug-in and the worst-case confidence intervals, supporting the same conclusions, viz. that possible correlations have little effect for our inference on the IV estimator.

For the confidence interval of the difference $\beta_{Diff}$from Eq. 3, the standard error in Eq. 4 can in principle be affected both through correlation between $\beta_{FTO\_TRAIT}$ and $\beta_{FTO\_BMI}$ (and consequently inflated ${se}_{IV}$) as well as through correlation between $\beta_{IV}$ and $\beta_{BMI\_TRAIT}$. Note, however that the latter is unlikely from the outset, because the standard error in Eq. 4 is dominated by ${se}_{IV}$, which is considerably larger than ${se}_{BMI\_TRAIT}$ (range of ratio ${se}_{IV}/{se}_{BMI\_TRAIT}$= 1.7-13.3, median of ratio = 5.5). Accordingly, the extra covariance term in the correlation-adjusted formula for the standard error

$${se}_{Diff}\text{=}\left. \sqrt{\left( {se}_{IV} \right)^{2}+\left( {se}_{BMI\_TRAIT} \right)^{2}+{se}_{IV} {se}_{BMI\_TRAIT} r} \right.$$

will have little effect on the total standard error, regardless of the strength of the correlation $r$ between $\beta_{IV}$ and $\beta_{BMI\_TRAIT}$.

This is also evident from Table S7 that presents the confidence intervals adjusted for the effect of both possible correlations: the plugin-estimate is based on using the between-cohort estimate of correlation for both $\beta_{FTO\_TRAIT}$ and $\beta_{FTO\_BMI}$, as well as $\beta_{IV}$ and $\beta_{BMI\_TRAIT}$. The worst case estimate is based on combining the worst-case scenarios for both standard errors. As in Table S6, we find that a) the possible effect of correlations is small, b) the plugin confidence intervals tend to be narrower than the reported confidence intervals, and c) even under the worst case scenario, we do not need to change our conclusions.

A small technical wrinkle in the derivation of the adjusted version of ${se}_{Diff}$ above is that Fieller's theorem does not provide an estimate for ${se}_{IV}$ per se, but only lower and upper confidence limits. We have side-stepped this issue by applying the correction factors based on the relative lengths of the confidence intervals listed in Table S6 to the standard errors based on Eq. 2.

According to Burgess and Thompson^[^[^11^](#_ENREF_11)^]^ some bias may be anticipated in the meta-analysis, compared to the analysis at individual level. In addition, *FTO* as a relatively weak instrument may also lead to biases. Therefore we have run extensive simulation studies to assess the bias and precision of the estimator in the settings of our study, by setting the association parameters between *FTO* and BMI to what has been observed here and varying the magnitude of the true causal effect.

As a result, quantitative trait analysis using the linear IV estimator did not lead to any biases, but for binary trait analysis, the Wald ratio led to somewhat biased estimates of the causal odds ratio. Realistically the bias can reach a maximum of 10 % for traits with high prevalence and greatest difference between the conventional BMI-trait association measure and the IV estimate, such as type 2 diabetes. However, given the fact that the IV estimate is further from the null value than the conventional estimate in this study for all dichotomous outcomes, the bias is always in the conservative direction: the true causal effect estimate is likely to be further from the null than the obtained IV estimate. Although there has been reported a possibility of bias away from the null in meta-analysis results, caused by the use of a large number of small studies, we were not able to reproduce any bias in a simulation study using the parameters corresponding to the observed effect sizes and sizes of cohorts involved in the present study.

In summary, the obtained IV estimates (Wald ratios) are less biased for the true causal effect between BMI and trait than the possibly confounded conventional BMI-trait association measures.

### References

1. Marchini J, Howie B, Myers S, McVean G, Donnelly P (2007) A new multipoint method for genome-wide association studies by imputation of genotypes. Nat Genet 39: 906-913.

2. Li Y, Willer CJ, Ding J, Scheet P, Abecasis GR (2010) MaCH: using sequence and genotype data to estimate haplotypes and unobserved genotypes. Genet Epidemiol 34: 816-834.

3. Higgins JP, Thompson SG, Deeks JJ, Altman DG (2003) Measuring inconsistency in meta-analyses. BMJ 327: 557-560.

4. Ioannidis JP, Patsopoulos NA, Evangelou E (2007) Uncertainty in heterogeneity estimates in meta-analyses. BMJ 335: 914-916.

5. Higgins JP, Thompson SG (2002) Quantifying heterogeneity in a meta-analysis. Stat Med 21: 1539-1558.

6. Riley RD, Higgins JP, Deeks JJ (2011) Interpretation of random effects meta-analyses. BMJ 342: d549.

7. Speliotes EK, Willer CJ, Berndt SI, Monda KL, Thorleifsson G, et al. (2010) Association analyses of 249,796 individuals reveal 18 new loci associated with body mass index. Nat Genet 42: 937-948.

8. NA S, S M, V D (2011) Mendelian Randomisation: A Tool for Assessing Causality in Observational Epidemiology. In: MD T, editor. Genetic Epidemiology Humana Press.

9. Palmer TM, Sterne JA, Harbord RM, Lawlor DA, Sheehan NA, et al. (2011) Instrumental variable estimation of causal risk ratios and causal odds ratios in Mendelian randomization analyses. American journal of epidemiology 173: 1392-1403.

10. Whitlock G, Lewington S, Sherliker P, Clarke R, Emberson J, et al. (2009) Body-mass index and cause-specific mortality in 900 000 adults: collaborative analyses of 57 prospective studies. Lancet 373: 1083-1096.

11. Burgess S, Thompson SG (2011) Avoiding bias from weak instruments in Mendelian randomization studies. Int J Epidemiol 40: 755-764.
